# Pore-forming moss protein bryoporin is structurally and mechanistically related to actinoporins from evolutionarily distant cnidarians

**DOI:** 10.1016/j.jbc.2022.102455

**Published:** 2022-09-03

**Authors:** Gašper Šolinc, Tomaž Švigelj, Neža Omersa, Tina Snoj, Katja Pirc, Nada Žnidaršič, Akiko Yamaji-Hasegawa, Toshihide Kobayashi, Gregor Anderluh, Marjetka Podobnik

**Affiliations:** 1 Department of Molecular Biology and Nanobiotechnology, National Institute of Chemistry, Ljubljana, Slovenia; 2 Department of Biology, Biotechnical Faculty, University of Ljubljana, Ljubljana, Slovenia; 3 Lipid Biology Laboratory, RIKEN, Wako-shi, Saitama, Japan; 4 UMR 7021 CNRS, Université de Strasbourg, Illkirch, France

**Keywords:** actinoporin, bryoporin, crystal structure, moss, *Physcomitrium (Physcomitrella) patens*, sphingomyelin, sphingolipids, sterols, stress response, transmembrane pores, CAMP, campesterol, CER, ceramide, CHOL, cholesterol, CPE, ceramide phosphoethanolamine, DOPC, 1,2-dioleoyl-*sn*-glycero-3-phosphocholine, ERG, ergosterol, GIPC, glycosylinositol phosphorylceramide, HALT, hydra's actinoporin-like toxin, LUV, large unilamellar vesicle, MLV, multilamellar vesicle, PDB, Protein Data Bank, PFP, pore-forming protein, RBC, bovine red blood cell, SM, sphingomyelin, SPR, surface plasmon resonance, TEM, transmission electron microscopy

## Abstract

Pore-forming proteins perforate lipid membranes and consequently affect their integrity and cell fitness. Therefore, it is not surprising that many of these proteins from bacteria, fungi, or certain animals act as toxins. While pore-forming proteins have also been found in plants, there is little information about their molecular structure and mode of action. Bryoporin is a protein from the moss *Physcomitrium patens*, and its corresponding gene was found to be upregulated by various abiotic stresses, especially dehydration, as well as upon fungal infection. Based on the amino acid sequence, it was suggested that bryoporin was related to the actinoporin family of pore-forming proteins, originally discovered in sea anemones. Here, we provide the first detailed structural and functional analysis of this plant cytolysin. The crystal structure of monomeric bryoporin is highly similar to those of actinoporins. Our cryo-EM analysis of its pores showed an actinoporin-like octameric structure, thereby revealing a close kinship of proteins from evolutionarily distant organisms. This was further confirmed by our observation of bryoporin’s preferential binding to and formation of pores in membranes containing animal sphingolipids, such as sphingomyelin and ceramide phosphoethanolamine; however, its binding affinity was weaker than that of actinoporin equinatoxin II. We determined bryoporin did not bind to major sphingolipids found in fungi or plants, and its membrane-binding and pore-forming activity was enhanced by various sterols. Our results suggest that bryoporin could represent a part of the moss defense arsenal, acting as a pore-forming toxin against membranes of potential animal pathogens, parasites, or predators.

Pore-forming proteins (PFPs) disrupt the normal functions of biological membranes by affecting their integrity and selective permeability. They are expressed by organisms of all kingdoms of life, with their biological roles spanning from involvement in immune response, development, digestion, and in most cases in attack or defense, thereby acting as toxins (1, 2, 3, 4, 5). PFPs are secreted by organisms as water-soluble monomers, which specifically or nonspecifically bind to either lipid, sugar, or protein components of the target membranes. The membrane-bound monomers then oligomerize into structured oligomers, followed by coordinated conformational changes in all protomers, resulting in formation of functional transmembrane pores. The transmembrane channels of these pores are either lined by symmetric α-helical clusters or β-barrels, based on which PFPs are classified into α- or β-PFPs (3, 6, 7, 8).

Among α-PFPs, actinoporins are one of the most studied families. They were first identified in sea anemones (Actiniaria) but were later found also in other cnidarians (8, 9, 10, 11), serving in defense against predators or aiding in prey capture (12, 13). Members of this family are small 20 kDa proteins and usually of a basic pI value (12, 14, 15). Most known actinoporins lack cysteine residues (16), except for the protein described in the coral *Stylophora pistilata*, containing a single cysteine (17). Members of actinoporin family share amino acid sequence identity of over 55% (18), resulting in a highly conserved 3D actinoporin fold (9, 10, 16, 19). Structures of several actinoporins have been reported to date, equinatoxin II (EqtII) from *Actinia equina* (20, 21), fragaceatoxin C (FraC), (22) and fragaceatoxin E (FraE) (23) from *Actinia fragacea*, as well as sticholysin I (StnI) (24) and sticholysin II (StnII) (19) from *Stichodactyla helianthus*. Monomeric actinoporins are built of a central β-sandwich of two parallel β-sheets, flanked by two α-helices. Actinoporins are known to bind to sphingomyelin (SM) head groups in lipid membranes, which are mediated by amino acid side chains from the central β-sandwich and the C-terminal α-helix (25, 26, 27). During the transition from membrane-bound oligomers to the transmembrane pore, the N-terminal amphipathic α-helix of each protomer detaches from the central β-sandwich. During this process, this α-helix is elongated by the incorporation of additional N-terminal residues and crosses the lipid membrane. As several protomers are simultaneously involved in transition, this consequently results in formation of a transmembrane channel walled by α-helices, intertwined by lipid molecules of the target membrane (22, 26, 28, 29, 30). Various stoichiometries of actinoporin pores have been suggested, from tetramers, hexamers, octamers to nonamers (8, 22, 31, 32, 33). To date, only one high resolution actinoporin pore structure has been determined, the octameric FraC pore (26).

Structural features of actinoporins have been found in a larger group of proteins despite their limited sequence identity to actinoporins and have been classified as actinoporin-like proteins (ALPs) (9, 34, 35, 36). ALPs are abundant in cnidarians, for example the hydra's actinoporin-like toxins (HALTs) (37, 38), in various molluscs (39, 40, 41), bacteria (42), oomycetes (36), fungi (35, 43), fish (9), as well as in plants (9). The function of toxic cnidarian ALPs, such as HALTs, is similar to that of actinoporins (37, 38). In fishes (9) and fungi (35, 44), it was proposed that they are involved in target cell recognition, while in oomycetes they act as virulence factors (45). In plants, it was particularly interesting that ALP sequences were detected in drought stress expressed sequence tag libraries from the mosses *Physcomitrium patens* (until recently known as *Physcomitrella patens*) (46), *Tortula ruralis* (47), and the lycophyte *Selaginella lepidaphylla* (48). Among these ALPs, bryoporin from *P. patens* was shown to share the highest amino acid sequence identity with actinoporins (Fig. S1) (9) and interestingly also the hemolytic activity that was inhibited by the specific actinoporin receptor SM (49). Furthermore, bryoporin was shown to take part in the moss stress response, as it was found upregulated by various abiotic stresses, especially by dehydration. The drought tolerance in *P. patens* was significantly increased upon overexpression of the bryoporin gene (49). Interestingly, the expression of the bryoporin gene was also shown to be upregulated during infection by the necrotrophic fungus *Botrytis cinerea* (50). These results suggested that ALPs could play important physiological role also in early land plants, especially during stress.

Here, we provide detailed description of the 3D structure and mechanism of action of the moss cytolysin bryoporin, revealing its close kinship with actinoporins from evolutionarily distant organisms. The mechanism of transmembrane pore formation and its architecture seems to be preserved despite the phylogenetic distance between sea anemones and mosses, including bryoporin’s preferential binding to lipid bilayers mimicking animal membranes, but not those of fungi or plants. Although the physiological role of bryoporin in the moss still remains to be resolved, our results suggest that this protein could act as a pore-forming toxin, targeting membranes of pathogens, parasites, or predators of an animal origin.

## Results

### The monomeric form of bryoporin has a typical actinoporin fold

**Figure 1:**
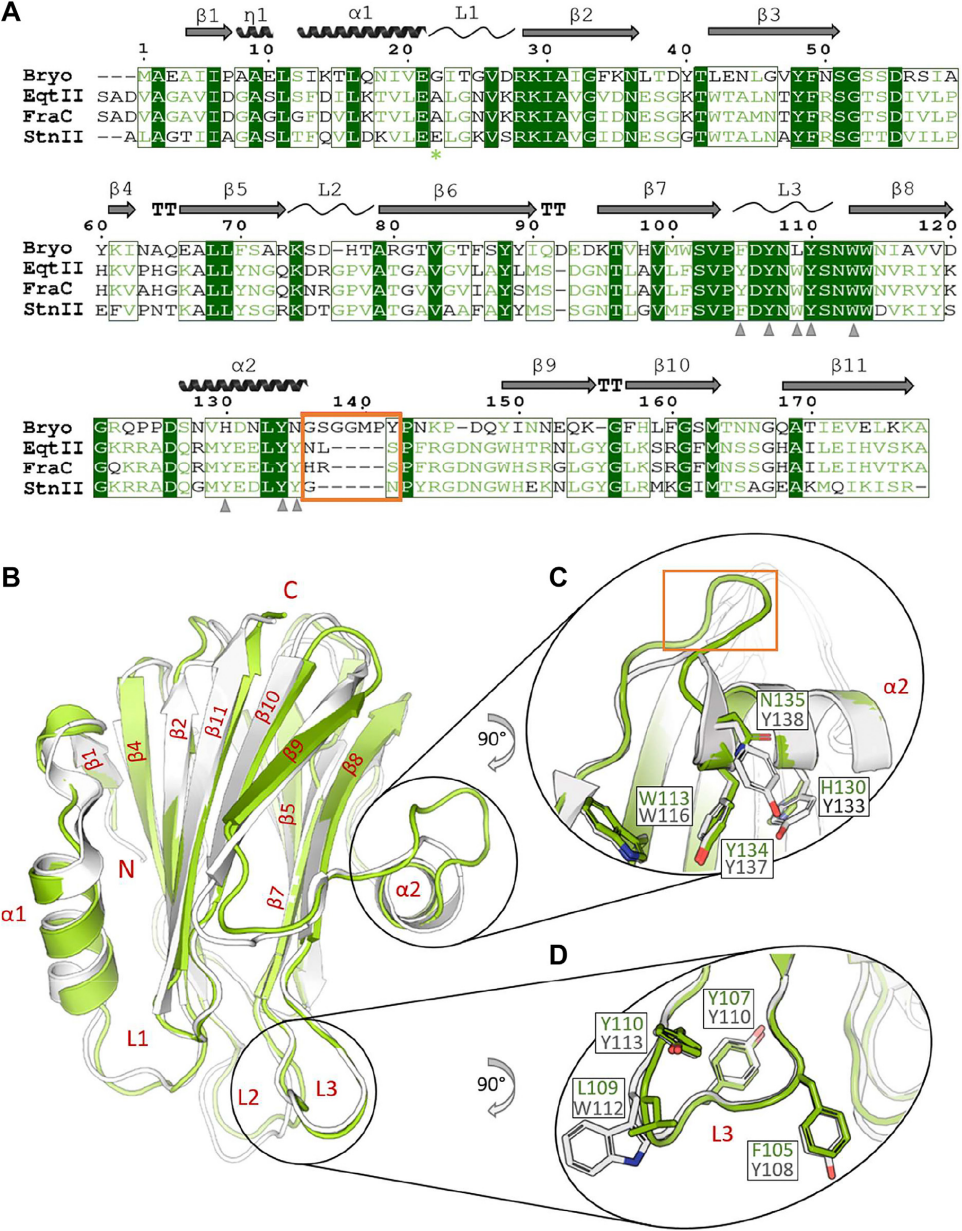
**Structural features of bryoporin.***A*, structure-based amino acid alignment of bryoporin with typical actinoporins. Bryoporin (Bryo; this work, PDB-ID 7PUD), EqtII (PDB-ID 1IAZ), StnII (PDB-ID 1GWY), and FraC (PDB-ID 3ZWG). Structures were aligned using PDBeFold (52). Schematic representation of bryoporin secondary structure (based on the structure of the monomeric bryoporin determined in this work) is presented above the sequence alignment. The *orange rectangle* marks an insertion of four additional amino acids present in the bryoporin sequence. Identical residues are marked with *green background* and chemically similar residues are displayed in *green font*, while chemically significantly different amino acid residues are indicated by *black letters*. The counting above the alignment is based on bryoporin. *Gray arrowheads* mark the aromatic amino acids that are conserved in actinoporins’ membrane-binding region (8, 26). *Green asterisk* marks the C-terminal end of the sequence used to model the transmembrane helix in the pore model (see Fig. 6, *D* and *E*). *B*–*D*, superposition of crystal structures of bryoporin (this work, PDB-ID 7PUD, *green*) and actinoporin EqtII (PDB-ID 1IAZ, *gray*). N and C termini and secondary structural elements are labeled by *red letters*. Two areas of the lipid-binding region are highlighted and zoomed in (*C*) and (*D*) panels. Side chains of residues involved in SM binding (8, 26) are shown in sticks. The *orange rectangle* in panel (*C*) marks the additional loop in bryoporin, which is also marked in (*A*). PDB, Protein Data Bank; SM, sphingomyelin.

Earlier studies suggested that bryoporin is structurally and functionally related to actinoporins (9, 49). Its polypeptide chain of 178 amino acid residues and molecular weight of approximately 20 kDa lacks cysteines and shares approximately 35% amino acid identity and additional 45% amino acid similarity with actinoporins EqtII, StnII, and FraC (Figs. 1*A* and S1). However, in contrast to typically basic pI values of most actinoporins (12, 51), bryoporin’s theoretical pI is at acidic pH value of 5.3 (http://www.expasy.org/tools/, accessed February 6, 2021).

To address how these known relations to cnidarian actinoporins are reflected in the 3D structure of bryoporin, we determined its crystal structure. Recombinant bryoporin (Fig. S2) crystallized in the space group P22121 with one molecule in the asymmetric unit. The structure of the monomeric bryoporin was determined at 1.25 Å resolution using a truncated structure of FraC (Protein Data Bank [PDB]-ID 3ZWJ) as a search model in molecular replacement procedure (Table S1). The electron density map was of high quality (Fig. S3) and fit all residues of the bryoporin polypeptide chain, except for the first three N-terminal residues. In addition, 13 residues of the C-terminal linker, containing the affinity His_6_-tag introduced during the cloning, could not be traced in the electron density, likely due to flexibility.

The overall structure of the monomeric bryoporin reveals a conserved actinoporin fold, thereby reflecting bryoporin’s high amino acid sequence similarity to actinoporins. Bryoporin is built of a compact β-sandwich composed of two parallel β-sheets of five and four β-strands, flanked by two α-helices (Fig. 1*B*). The RMSD between bryoporin and actinoporin EqtII (PDB-ID 1IAZ) for aligned 169 Cα atoms is 0.99 Å (Fig. 1*B*) (PDBeFold (52)). For comparison, the RMSD value between sea anemone actinoporins EqtII and FraC (PDB-ID 3ZWG), which share 90% of identical amino acids, is 0.49 Å (174 Cα atoms aligned). The only notable deviation from the typical actinoporin fold is the additional four-residue loop between the C-terminal α2-helix and β9-strand (Fig. 1, *A* and *C*). Interestingly, while similar loops are not present in any known actinoporin structure, a loop of the same length at the equivalent position is present in HALT-1 (PDB: 7EKZ) (53), with which bryoporin shares 25% amino acid identity and additional 39% similarity, with the RMSD of 1.53 Å. Unlike in HALT-1, where α2-helix is shorter for four amino acids, in bryoporin, this helix is of the same length as in the sea anemone actinoporins (Fig. S4).

The structure of bryoporin harbors a patch of surface exposed hydrophobic amino acids in the L3 loop and α2-helix that are spatially close to each other and conserved in actinoporins (Fig. 1, *A*, *C*, and *D*), where they play a crucial role in membrane binding (8, 29). The residues that are involved in SM binding in actinoporins are also conserved in the structure of bryoporin (Figs. 1 and S5) (8). For example, Tyr110, Tyr113, Trp116, and Tyr137 of EqtII have their counterparts Tyr107, Tyr110, Trp113, and Tyr134 in bryoporin (Fig. 1, *A*, *C*, and *D*). The exceptions are at position of Tyr108 and Trp112 of EqtII, which are replaced by Phe105 and Leu109 in bryoporin, respectively. However, such substitutions have also been identified among actinoporins, for example, Tyr108 to Phe substitution was found in StnII (9, 19), and of Trp112 to Leu or Phe in magnificalysin from *Heteractis magnifica* and PsTX-20A from *Phylodiscus semoni* (25, 54, 55). Replacement of Trp112 with Leu in EqtII did not affect SM specificity and membrane-binding ability (25, 56). Furthermore, the counterparts of Tyr133 and Tyr138 of EqtII in bryoporin are His130 and Asn135, respectively (Figs. 1, *A* and *D* and S5). Again, the replacement of Tyr133 (EqtII numbering) with a more polar residue, such as Ser or His, has been reported for some actinoporins (18), and among ALPs, this position can be occupied even by Glu (9). Replacement at the position of Tyr138 (EqtII numbering) with Asn is the second most common in other actinoporins (9, 18). An additional notable difference between bryoporin and a typical actinoporin, such as FraC, is in the electrostatic surface potential, which is overall more negative in the case of bryoporin (Fig. S6). Importantly, despite this difference, the charge in lipid-binding region is comparable between bryoporin and FraC (Fig. S6). In summary, the moss protein bryoporin shares high structural similarity with pore forming actinoporins from evolutionarily distant organisms, at the amino acid level as well as in the 3D fold.

### Bryoporin is a PFP with specificity for SM-containing membranes

**Figure 2:**
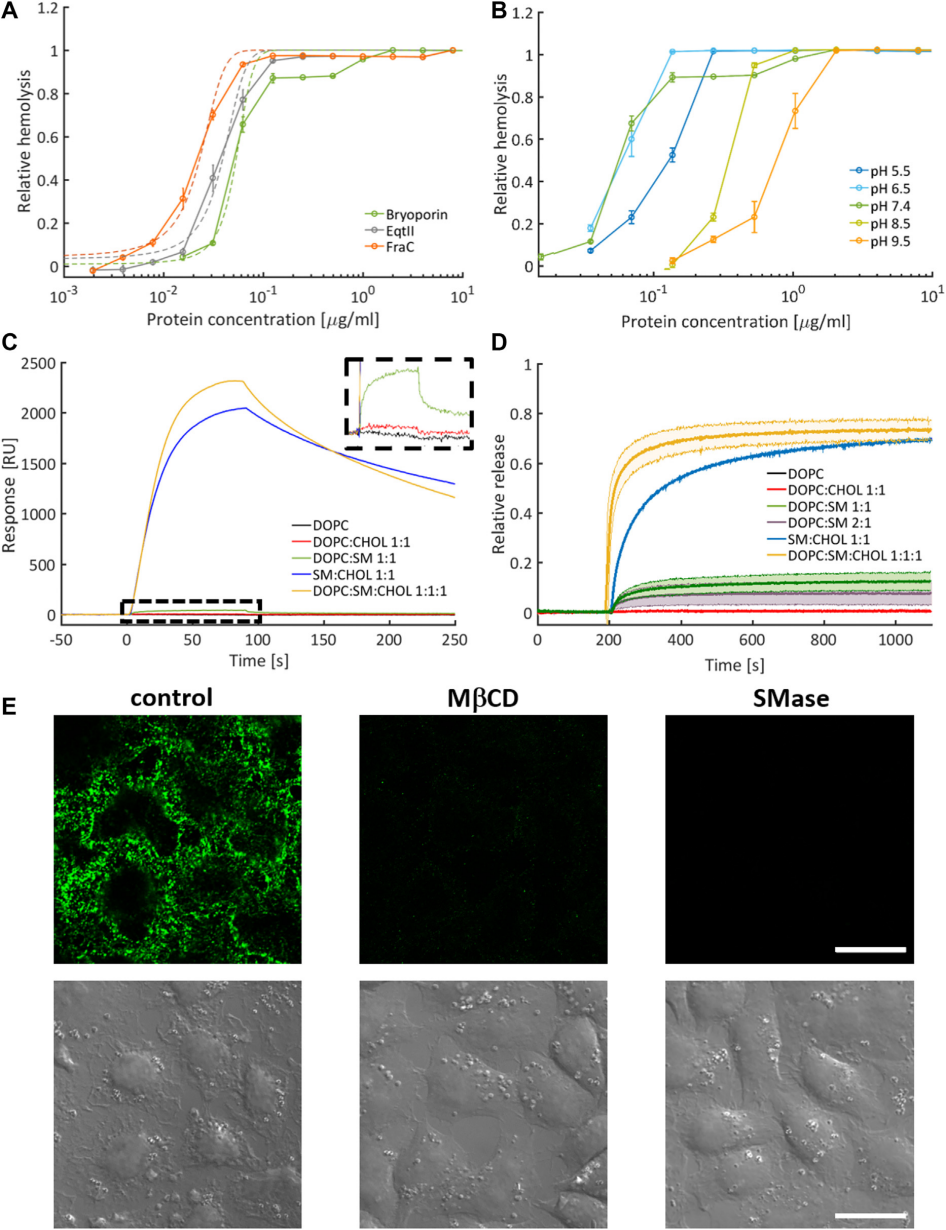
**Interaction of bryoporin with model lipid membranes.***A*, comparison of hemolytic activity of bryoporin, EqtII, and FraC at pH 7.4. The *dashed lines* are fits of the logistic function and the midpoint was used to estimate EC_50_ (Table 1). *B*, hemolytic activity of bryoporin at different pH values. (*A* and *B*) show relative hemolysis (*i.e.*, change in absorbance at 630 nm normalized to 1 after 20 min). Each measurement was repeated three times. Points represent mean value ± SD. *C*, SPR measurements of 500 nM bryoporin binding to lipid bilayers of various lipid compositions. The inset shows zoomed area, as marked. *D*, kinetics of calcein release from 20 μM LUVs of different lipid compositions induced by 100 nM bryoporin. The *bold central line* is an average of three measurements, *shaded area* shows SD. *E*, bryoporin binding to HeLa cells. 5 μg/ml of bryoporin was added to nontreated (control, *left panel*) cells or cells pretreated with 20 mM MβCD (*central panel*) or 1.7 unit/ml SMase (*right panel*). Bryoporin was detected by fluorescently labeled antibody. Differential interference contrast images corresponding to each panel are shown below. The scale bar represents 20 μm. LUV, large unilamellar vesicle; SPR, surface plasmon resonance.

To show how the high structural resemblance of bryoporin with actinoporins is reflected in its mechanism of action, we first compared the dose-dependence of the bovine red blood cell (RBC) hemolysis caused by bryoporin with the sea anemone actinoporins EqtII and FraC (Fig. 2*A*). The hemolytic activity of all three proteins showed a clear sigmoidal dependence on the protein concentration. While at pH 7.4 bryoporin displayed the lowest hemolytic activity, with EC_50_ of 2.7 nM, its activity was not drastically different from EqtII and FraC, with EC_50_ 2 nM and 1.2 nM, respectively (Fig. 2*A* and Table 1). Bryoporin’s hemolytic activity is optimal at neutral and slightly acidic pH and drops remarkably at more acidic and basic pH values, 2-fold and 10- to 20-fold, respectively (Fig. 2*B* and Table 1).

**Table 1:** Half maximal effective concentration (EC_50_) of hemolytic activity of bryoporin, EqtII and FraC, estimated as the midpoint of logistic function fitted to data presented in Figure 2, *A* and *B*

Protein	EqtII	FraC	Bryoporin				
pH	7.4	7.4	5.5	6.5	7.4	8.5	9.5
EC_50_ (nM)	2.0 ± 0.0032	1.2 ± 0.0008	6.1 ± 0.0014	2.9 ± 0.00025	2.7 ± 0.0034	16.7 ± 0.0023	39.6 ± 0.0026

Hemolytic activity of bryoporin was shown to be inhibited by SM, a specific actinoporin membrane receptor (49). To assess bryoporin’s preference for SM, we performed surface plasmon resonance (SPR) experiments using lipid membranes of different compositions. Bryoporin did not bind to membranes composed of pure 1,2-dioleoyl-*sn*-glycero-3-phosphocholine (DOPC) or mixture of DOPC and cholesterol (CHOL) (DOPC:CHOL, molar ratio 1:1) (Fig. 2*C*), while weak interaction was observed in the case of DOPC:SM (molar ratio 1:1) (Fig. 2*C* inset). However, bryoporin bound to the SM:CHOL membrane (molar ratio 1:1), and the binding was even more pronounced when the three component membrane system DOPC:SM:CHOL (molar ratio 1:1:1) was used (Fig. 2*C*). To test how this complies with the pore forming activity of bryoporin, we performed the calcein release assay using large unilamellar vesicles (LUVs) of the same membrane compositions as for binding studies (Fig. 2*D*). As expected, bryoporin did not damage DOPC or DOPC:CHOL (molar ratio 1:1) vesicles. Addition of SM favored pore formation, as ∼10% and ∼20% of calcein was released from 2:1 and 1:1 DOPC:SM LUVs, respectively (Fig. 2*D*). However, calcein release greatly increased when both, SM and CHOL, were present in the membrane and in agreement with SPR measurements, was the highest in the case of a three component membrane system of DOPC:SM:CHOL, reaching 70% of the maximum release.

We next examined the bryoporin binding to SM and CHOL in natural membranes of HeLa cells. Bryoporin bound to untreated cells (Fig. 2*E*). Its binding was notably decreased, although not completely abolished, upon the pretreatment of cells with the CHOL-depleting reagent methyl-β-cyclodextrin (MβCD) and completely absent when cells were pretreated with the SM phosphodiesterase (SMase), which cleaves SM in the cell membrane. Altogether, our results show bryoporin’s specificity to SM in target membranes. In addition, membrane binding and consequently pore formation are enhanced by CHOL, which further suggests that bryoporin’s mechanism of action closely resembles that of actinoporins (57, 58, 59).

### Various sterols enhance bryoporin’s pore-forming activity

**Figure 3:**
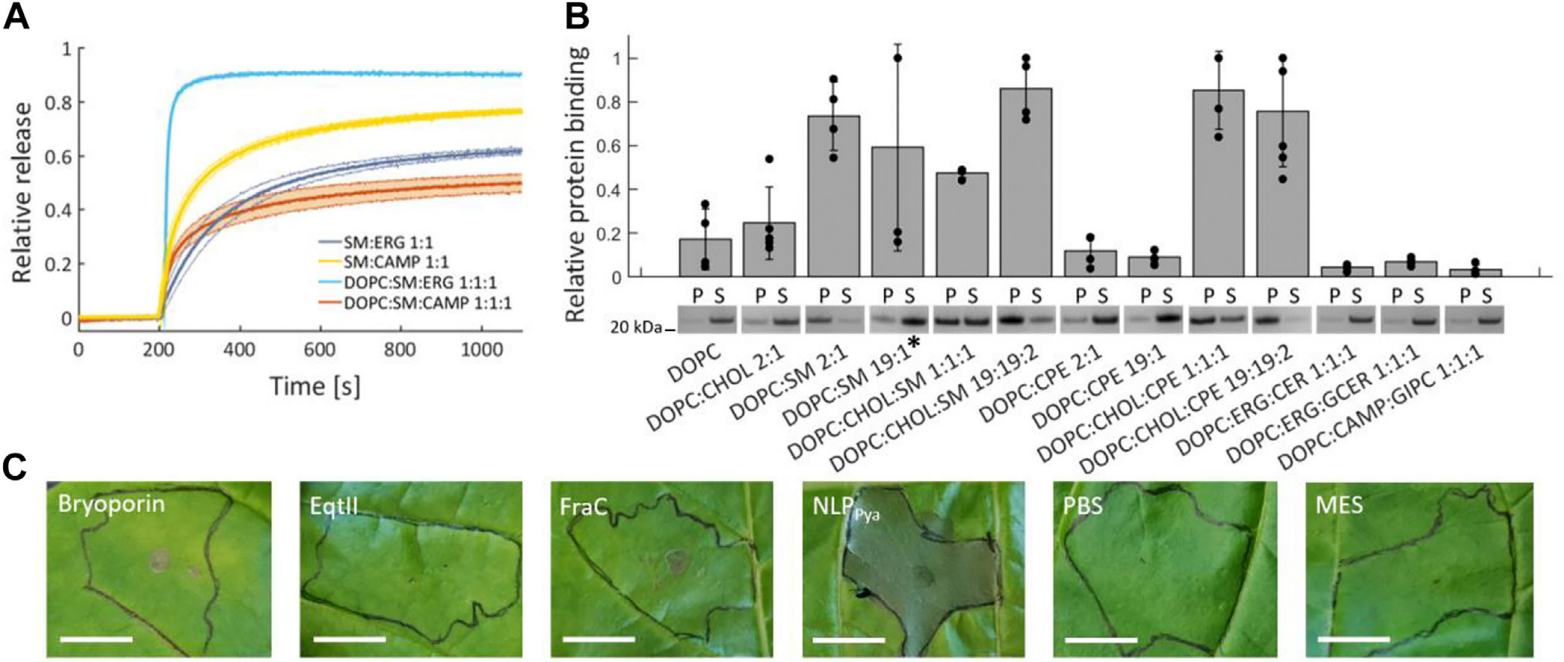
**Bryoporin’s pore forming activity and binding to various lipid membranes.***A*, kinetics of calcein release induced by 100 nM bryoporin from 20 μM LUVs containing campesterol (CAMP) or ergosterol (ERG). The *bold central line* is the average of three measurements, the *shaded area* shows SD. *B*, SDS-PAGE analysis of bryoporin binding to multilamellar vesicles as determined by the lipid sedimentation assay. All lipid compositions are in molar ratios. The presence of a protein band in the pellet fraction indicates binding. The ratio between P and S bands was quantified. Vesicles with different lipid ratios were used. Molar ratios 19:1 in DOPC:SM/CPE and 19:19:2 in DOPC:CHOL:SM/CPE correspond to 5% SM or CPE in membranes. N = 4 to 6, relevant cut-out parts of gels are shown. Images of the representative gels are shown in Fig. S7. *C*, necrotic lesions formation on tobacco leaves. Leaves were infiltrated with 10 μM solutions of bryoporin, EqtII, FraC in PBS, and NLP_Pya_ in Mes buffer. Infiltrated area is marked. The *upper surface* of the leaf was photographed after 48 h. N = 3. The scale bar represents 1 cm. All replicates are shown in Fig. S8. CPE, ceramide phosphoethanolamine; DOPC, 1,2-dioleoyl-*sn*-glycero-3-phosphocholine; LUV, large unilamellar vesicle; P, pellet; S, supernatant; SM, sphingomyelin.

We then tested whether the enhancement of bryoporin activity is sterol specific. We performed the calcein release experiments with LUVs, where CHOL, a typical animal sterol, was replaced with campesterol (CAMP) or ergosterol (ERG), commonly found in plants or fungi, respectively (60, 61) (Fig. 3*A*). In both cases, bryoporin successfully released calcein from vesicles. In the case of ERG the response was similar as in the case of CHOL (Fig. 2*D*), with calcein release notably higher for the three component system (DOPC:SM:ERG) than for two component system (SM:ERG). Interestingly, while CAMP also efficiently enhanced bryoporin-induced membrane damage, the results were inverse, with higher pore-forming activity of bryoporin in the two component system, SM:CAMP (Fig. 3*A*). These results show that sterols enhance the pore-forming activity of bryoporin. This feature is common among sterols and could be caused by the effect of sterols on membrane fluidity and/or lipid organization, thereby favoring successful pore formation.

### Bryoporin binds to animal but not to fungal or plant sphingolipid receptors

SM, a type of sphingolipid found in animal cell membranes (62), is the only known lipid receptor for actinoporins (16, 25). To test whether the plant actinoporin bryoporin exhibits broader specificity for sphingolipids, we analyzed its binding to multilamellar vesicles (MLVs) prepared with different sphingolipids in combination with sterols to mimic membranes of different types of organisms (Figs. 3*B* and S7). Vesicles with SM and CHOL were used as a general model for the animal membrane, and ceramide phosphoethanolamine (CPE) in combination with CHOL was used as a model of the invertebrate membrane (63). Fungal and plant sphingolipids are very diverse. Nevertheless, we used ceramide (CER) and glucosylceramide (GlcCER) (64) in combination with ERG to model the fungal membrane (65) and glycosylinositol phosphorylceramides (GIPCs) isolated from tobacco leaves in combination with CAMP as a model of plant membrane (65, 66). As expected, bryoporin bound to vesicles containing SM and to those containing both, SM and CHOL. In the case of DOPC:CHOL:SM 1:1:1, the protein was present also in the supernatant fraction, which could be attributed to poor sedimentation of disrupted vesicles, as other methods confirmed strong binding to this type of membrane (Fig. 2, *C* and *D*). Interestingly, bryoporin bound completely to vesicles containing 5% SM when CHOL was present (DOPC:CHOL:SM, molar ratio 19:19:2), while the protein binding varied largely in the absence of CHOL (DOPC:SM molar ratio 19:1). While bryoporin did not bind to CPE containing membranes in the absence of CHOL, the binding to membranes was significantly increased upon addition of CHOL, at 33% or 5% of CPE (DOPC:CHOL:CPE molar ratio 1:1:1 or 19:19:2, respectively) (Fig. 3*B*). About 5 mol percent of CPE in membranes was used as this is the physiologically relevant concentration of CPE in insect membranes (63). Bryoporin did not bind to membranes containing sphingolipids CER, GlcCER, or GIPCs found in fungal or plant membranes, including moss *P. patens* (67). To check whether bryoporin has any toxic activity toward plants, we infiltrated tobacco leaves with bryoporin, EqtII and FraC, and plant tissue necrotic ALP NLP_Pya_ as a control (36, 68) (Figs. 3*C* and S8). Bryoporin, EqtII and FraC, did not induce tobacco leaf necrosis at the concentration of 1 μM (Fig. S8) or 10 μM (Fig. 3*C*), while the same concentration of NLP_Pya_ caused a notable damage to the infiltrated leaves.

**Figure 4:**
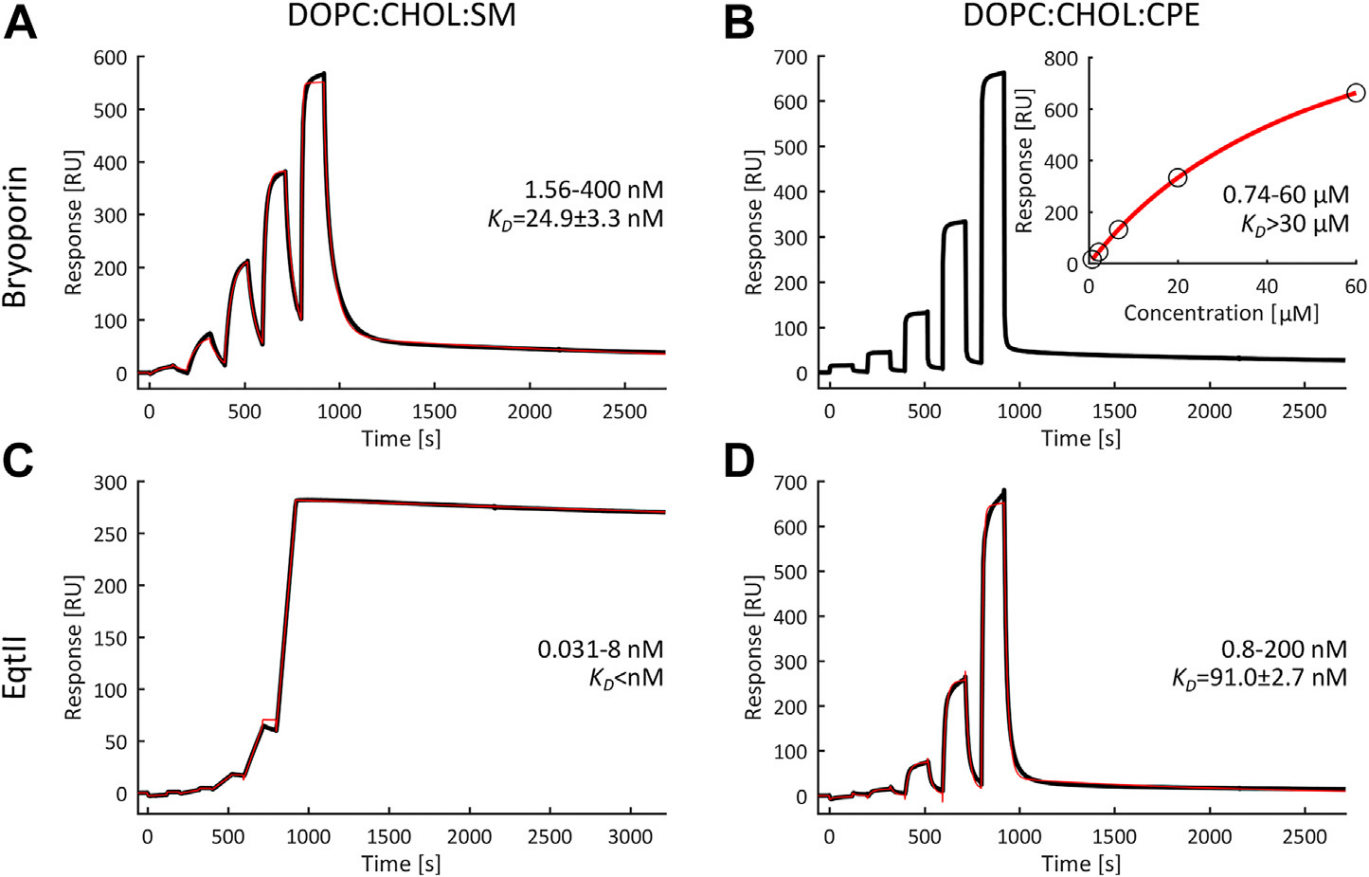
**Kinetic analysis of bryoporin and EqtII interactions with SM or CPE containing lipid membranes.** Lipid membranes were composed of DOPC:CHOL:SM/CPE (19:19:2 M ratio). Protein concentration ranges and *K*_*D*_ values are indicated. Double referenced sensorgrams (*black lines*) and two-state interaction kinetic fits (*red lines*) are shown in (*A*), (*C*), (*D*), and steady-state affinity fit is shown in (*B*). The derived kinetic constants are reported in Table S2. CHOL, cholesterol; CPE, ceramide phosphoethanolamine; DOPC, 1,2-dioleoyl-*sn*-glycero-3-phosphocholine; SM, sphingomyelin.

Next, we quantitatively analyzed the binding of bryoporin to SM and CPE containing membranes and compared it with the well-studied actinoporin EqtII (Fig. 4 and Table S2) (69, 70). Membranes with the lipid composition DOPC:CHOL:SM (or CPE) with 19:19:2 molar ratio were used. As expected, SPR analysis showed strong interactions of bryoporin with the DOPC:CHOL:SM membrane, with a *K*_*D*_ of 24.85 ± 3.33 nM (Fig. 4*A* and Table S2). EqtII bound even stronger to the SM-containing membrane, with a very fast association and extremely slow dissociation rate, resulting in a subnanomolar value of *K*_*D*_ (Fig. 4*C* and Table S2). Bryoporin and EqtII also bound to DOPC:CHOL:CPE (19:19:2) membranes but with much lower affinity. The *K*_*D*_s were an order of magnitude higher for bryoporin, >30 μM, and 91.0 ± 26.7 nM for EqtII (Fig. 4, *B* and *D* and Table S2). Overall, our results indicate that bryoporin binds preferentially to membranes containing sphingolipids such as SM and CPE, which are typically present in animal plasma membranes; however, its binding affinity to both types of membranes is significantly lower than that of EqtII.

### Bryoporin forms homogeneous and stable ion-conducting pores on planar lipid membranes

**Figure 5:**
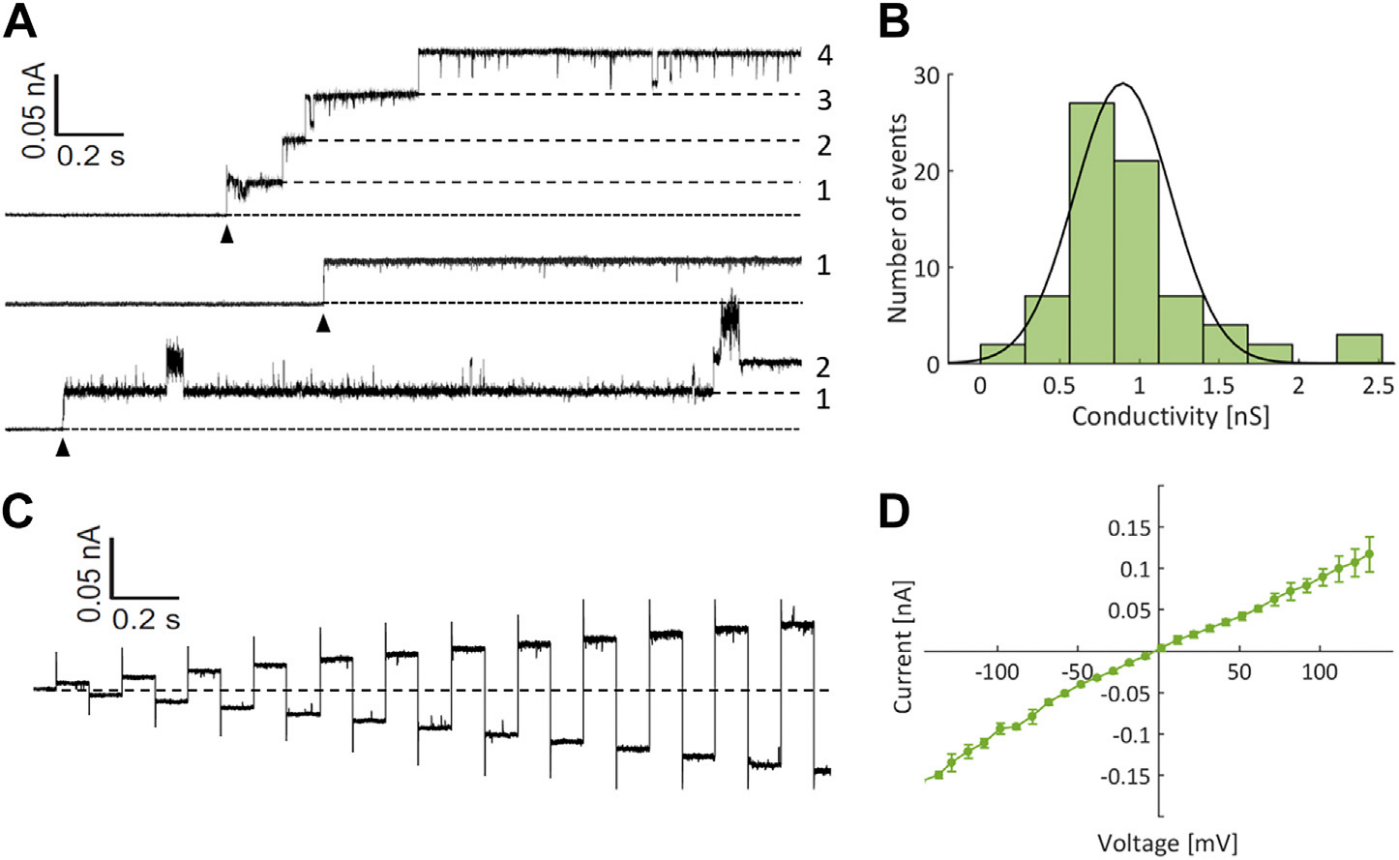
**Planar lipid bilayer experiments with bryoporin.***A*, examples of a planar lipid membrane (DOPC:SM, 1:1 M ratio) current trace, I (nA), *versus* time (s), at a constant applied voltage of 50 mV in 0.5 M NaCl, 20 mM Mes, pH 6. Bryoporin (51 nM) was added to the *cis* chamber. *Lines* and *numbers* mark individual pore insertions. *Arrows* point at the insertion of the first pore. *B*, single-channel conductance histogram of pores formed by bryoporin at +50 mV in 0.5 M NaCl, 20 mM Mes, pH 6. The line shows a normal distribution fit to the data (excluding four outliers) with a mean of 0.9 nS and a SD of 0.3 nS. *C*, single pore current trace at different voltages (alternating positive and negative steps of 5 mV in the range of −60 to 60 mV). *D*, ion-voltage (IV) curve of single bryoporin pore, recorded at the same conditions as in (*A*). The data points are an average of five measurements, error bars show the standard error. DOPC, 1,2-dioleoyl-*sn*-glycero-3-phosphocholine; SM, sphingomyelin.

We further characterized pores formed by bryoporin by using planar lipid membrane system to assess conductance of the ionic current through the pores. Several seconds after the addition of monomeric bryoporin to the *cis*-side of the planar lipid bilayer composed of DOPC and SM in 1:1 M ratio, we observed step-like increases in the current of well-defined amplitude (Fig. 5*A*). After pore formation, the pores were stable as we did not observe any pores closures (Fig. 5*A*). The mean conductance at 50 mV was 0.9 ± 0.3 nS (Fig. 5*B*), which is comparable to the conductance of EqtII and FraC actinoporin pores (14, 71). Pore conductance was homogeneous indicating a single stoichiometry (72). As expected, no pore formation was detected on membranes composed of pure DOPC or 1,2-diphytanoyl-*sn*-glycero-3-phosphocholine (DPhPC) (data not shown). The current at different voltages (Fig. 5*C*) and the corresponding IV curve (Fig. 5*D*) of bryoporin pore show slight nonlinearity as the pore is more conductive at negative voltages compared to equivalent positive voltages. This shape of the curve (Fig. 5*D*) is similar to that of FraC (72) and implies similar pore geometry.

### Bryoporin forms octameric transmembrane pores of the same architecture as actinoporins

**Figure 6:**
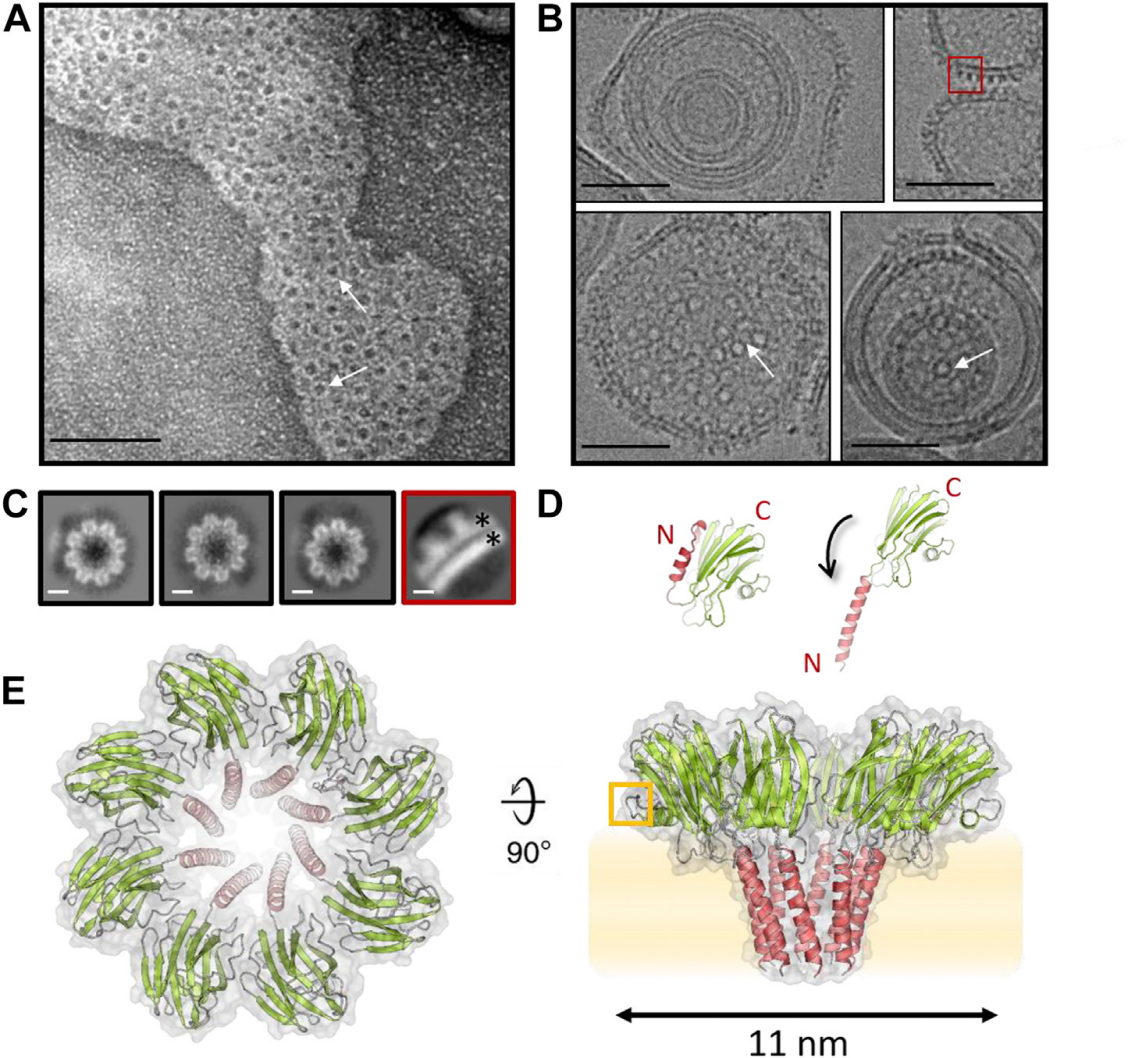
**Electron microscopy (EM) of bryoporin pores and the three-dimensional model of the pore.***A*, negative stain TEM micrograph of DOPC:SM:CHOL (molar ratio 1:1:1) MLVs incubated with bryoporin. The scale bar represents 50 nm. *B*, examples of DOPC:SM:CHOL (molar ratio 1:1:1) MLVs incubated with bryoporin recorded with cryo-EM. The scale bar represents 50 nm. *White arrows* point at examples of pores (top views). The *red rectangle* highlights a side view of the pore embedded in the membrane. *C*, 2D classes of bryoporin pores. Particles were picked from cryo-EM micrographs. Highlighted is the class of a side view of the pore embedded in the membrane. *Black asterisks* mark the two bilayers of the membrane. The scale bar represents 4 nm. *D*, *left*, structure of the bryoporin monomer, *right*, a model of the bryoporin protomer in the pore. The N-terminal region used to model the transmembrane helix is shown in *red* for both cases. N and C termini are marked by *red letters* N and C. *E*, cartoon representation of a model of bryoporin octameric pore (*green* and *red*) and pore molecular surface in *gray*. The pore model is based on the known crystal structure of octameric FraC pore (PDB-ID 4TSY). *Left*, top view of the pore; *right*, side view of the pore positioned in the lipid membrane (*shaded orange area*). The *orange rectangle* highlights the loop unique to bryoporin. CHOL, cholesterol; DOPC, 1,2-dioleoyl-*sn*-glycero-3-phosphocholine; MLV, multilamellar vesicle; PDB, Protein Data Bank; SM, sphingomyelin; TEM, transmission electron microscopy.

All results to this point suggested high similarity of bryoporin to sea anemone actinoporins, at the structural and mechanistic level. Furthermore, the planar lipid membrane experiment suggested that the stoichiometry of bryoporin transmembrane pores is highly homogeneous. To confirm this, we mixed the monomeric bryoporin with MLVs composed of DOPC:SM:CHOL (molar ratio 1:1:1) and imaged the vesicles with negative staining transmission electron microscopy (TEM) (Fig. 6*A*) and cryo-EM (Fig. 6*B*). Two clearly distinguishable pore orientations were observed on the micrographs, ring-shaped top views, present both in negative staining TEM and cryo-EM images (white arrows in Fig. 6, *A* and *B*), and pore side views, only visible on cryo-EM images (marked with a red rectangle in Fig. 6*B*). Thousand pore particles were picked to generate 2D class averages of top views of the pores imaged by cryo-EM (Fig. 6*C*). These 2D classes revealed that the bryoporin pore embedded in the lipid bilayer is ring shaped and built of eight protomers, with an outer diameter of 11 nm. No other pore stoichiometries were observed.

**Figure 7:**
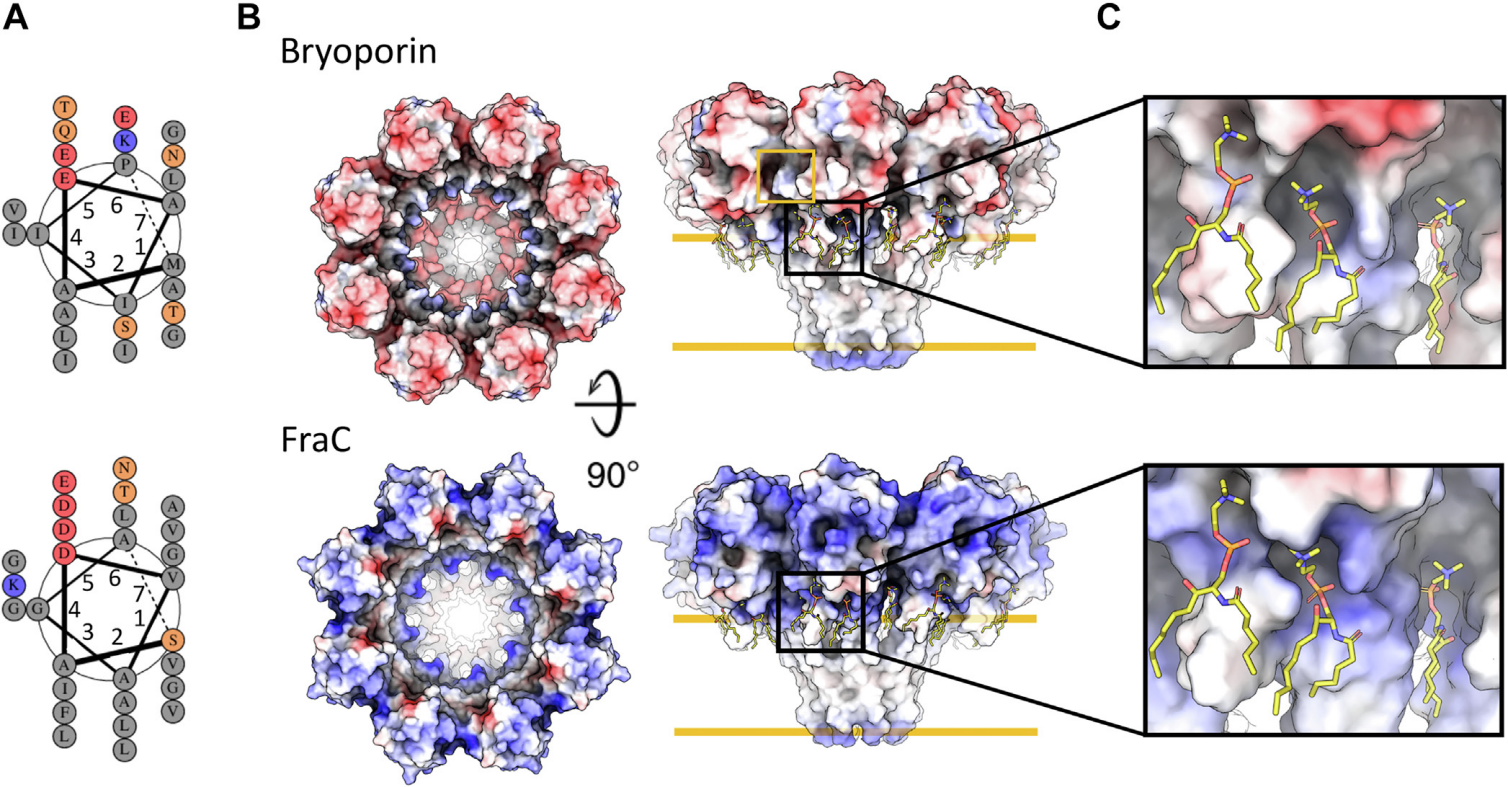
**Comparison of the modeled bryoporin pore and the crystal structure of FraC transmembrane pore.***A*, helical wheel diagram of transmembrane helices of modeled bryoporin pore and FraC pore. *B*, electrostatic surface potentials calculated with APBS plugin in PyMol (95) for bryoporin pore model (*top*) and FraC (*bottom*), top and side views are shown (*red* −5 kbT/ec, *blue* +5 kbT/ec). The *orange rectangle* highlights the additional loop present in bryoporin. *Orange lines* mark the lipid membrane. *C*, close-up of the area where phosphorylcholine molecules are bound in the FraC pore (PDB-ID 4TSY) and equivalent position on bryoporin. The phosphorylcholine moieties (*sticks*) were modeled into the bryoporin pore model according to the FraC structure. PDB, Protein Data Bank.

These results suggested high resemblance of the bryoporin pore architecture to the crystal structure of actinoporin FraC pore (PDB-ID 4TSY) (26), based on which we prepared a model of the bryoporin pore. To achieve this, we modeled the transmembrane N-terminal α-helix of bryoporin up to Gly25 (Figs. 1 and 6*D*) using the FraC pore as a template, while the core (β-sandwich and C-terminal α-helix) of the bryoporin protomer (Fig. 6*D*) remained unchanged. Transmembrane helices of both model bryoporin pore and FraC pore are amphipathic, with a hydrophobic and hydrophilic side of the helix as seen in the helical wheel diagram (Fig. 7*A*). We then superimposed eight bryoporin protomers on the corresponding FraC pore structure with no clashes originating from the bryoporin protomers. The outer diameter of approximately 11 nm of such a model corresponds to the one measured in 2D classes (Fig. 6, *C* and *E*).

While these results indeed show high resemblance of the bryoporin pores to actinoporin pores, we observed a notable difference in the electrostatic potential of the surfaces between the proposed model of the bryoporin pore and that of FraC (Fig. 7*B*). In the case of the bryoporin pore model, both outer cap of the pore as well as the inner surface of the pore is negatively charged (Fig. 7*B*). The area of phosphorylcholine binding, as suggested based on FraC pore structure, is positively charged, both in the case of bryoporin and FraC, with the phosphate of the phosphorylcholine head group located closer to the binding region and the choline group pointing away from the pore (Fig. 7*C*). The additional loop unique to bryoporin (Figs. 1 and 6*E* (right) and Fig. 7*B*) is, as suggested by the model, positioned above the region, where the pore comes in contact with the lipid head groups (Fig. 7, *B* and *C*) and does not seem to affect the shape of the pore channel. In summary, bryoporin forms transmembrane pores of homogeneous octameric stoichiometry of the same architecture as actinoporins but with a different distribution of surface electrostatic potential.

## Discussion

Genes encoding PFPs have been found in plants, however, with limited information on their mechanism of action and biological role. That actinoporin-like PFPs from the venoms of cnidarians and other aquatic animals, bacteria, and fungi act as toxins, is not surprising due to the life style of these organisms. On the other hand, the discovery of the stress-upregulated actinoporin-like gene in moss and the hemolytic activity of its protein product, bryoporin, was rather striking (9, 46, 49). Suggestions for biological roles of plant PFPs have been reported for a member of the aerolysin family of β-PFPs, FEM32, from the diecious plant *Rumex acetosa*, where its overexpression was found to alter flower development and induced male sterility in transgenic tobacco. The authors suggested this could be achieved by FEM32 forming pores in membranes, inducing a programmed cell death (73). Another example from this PFP family is enterolobin, from the seeds of the tropical tree *Enterolobium contortisiliquum*, which was shown to be cytolytic and toxic to the larvae of the beetle *Callosobruchus maculatus* (74).

To our knowledge, the structure and molecular mechanism of action of plant PFPs have not yet been experimentally described. To contribute to the understanding of molecular mechanisms behind the biological role of the actinoporin-like protein from moss, we performed a thorough characterization of bryoporin at the molecular level. Our study revealed a close structural and mechanistic kinship of bryoporin to pore-forming toxins actinoporins from evolutionarily distant aquatic organisms. Bryoporin not only shares high amino acid identity and similarity with sea anemone actinoporins but also highly conserved fold of the soluble monomer with a conserved membrane-binding region and quaternary structure of the transmembrane pores.

Interestingly, in our experimental setup, bryoporin highly preferred binding to membranes containing animal sphingolipid receptors, that is, SM and CPE, which was notably enhanced by the presence of sterols. SPR results revealed strong interactions of bryoporin and EqtII with membranes containing SM and an order of magnitude weaker but still significant interactions with membranes containing CPE (Fig. 4 and Table S2). Interestingly, the binding affinity of bryoporin was notably weaker than that of EqtII for membranes containing either SM or CPE. This difference between the two proteins reflects specific variabilities in amino acid composition and their spatial distribution (Figs. 1, S5 and S6). However, as discussed previously, the amino acid variability in the membrane-binding region can be found also among cnidarian actinoporins, where it is probably also responsible for slightly different affinities for the membranes observed among cnidarian actinoporins (75). Since bryoporin accumulates otherwise allowed amino acid substitutions, this results in a more profound effect on the electrostatic surface potential as well as on the shape of the sphingolipid-binding site (Figs. 6 and S6). However, as we show here, these differences do not affect the preference for SM or CPE nor do they affect the membrane-binding and pore-forming activity of bryoporin to an extent that is outside the expected range within the actinoporin family (75). On the other hand, bryoporin did not bind to CER, GlcCER, or GIPC containing membranes, which are representative sphingolipids in fungi and plants, including moss (64, 65, 67). Binding to SM and similarly shaped CPE is enabled by the architecture of the bryoporin lipid-binding site, where the phosphate groups of phosphocholine and phosphorylethanolamine fit into the positively charged binding region (Fig. 7*C*). CER and GlcCER lack this phosphate group, and although GIPCs contain it, additional sugars and thus a much bulkier GIPC head group (Fig. S9) likely present a steric hindrance for binding to bryoporin.

The 3D structure of the bryoporin monomer and the model of its transmembrane pore is, to our knowledge, the first detailed structural description of a plant cytolysin. High structural and mechanistic resemblance of bryoporin to actinoporins suggests that bryoporin could act as a pore-forming toxin to defend the moss against various predators and parasites of an animal origin. While no binding of bryoporin to fungal or plant mimicking lipid membranes could be observed, we cannot completely exclude interaction with fungal and plant membranes due to high variety of sphingolipids in these membranes and changes in their lipid composition that may occur during environmental stress (64, 67). As a low affinity interaction was shown for the sea anemone actinoporin FraC with carbohydrates (76), bryoporin could potentially also bind to sugar moieties on the target cell surface. Alternatively, bryoporin could be involved in processes, such as programmed cell death, as a response to abiotic stress (77) or play a role in a signaling pathway independently of its pore-forming ability. Finally, we also provide a structural model of a new actinoporin pore with unique surface properties. The bryoporin pore is homogeneous in its structure and stable in planar lipid membranes, which is crucial for applications such as the nanopore sensing, where the funnel-shaped actinoporin pores have been already successfully utilized (71, 72, 78, 79, 80).

## Experimental procedures

### Protein expression and purification

The bryoporin gene (49) (UniProt-Q5UCA8) was ordered from GenScript and subcloned into pET24a vector. The protein with C-terminal His_6_-tag was expressed in *Escherichia coli* BL21 (DE3). Bacterial cultures were grown in 1 l of terrific broth medium supplemented with 100 μg/ml ampicillin at 37 °C with shaking. When culture absorbance at 600 nm reached 0.6 to 0.8, protein expression was induced with 0.4 mM IPTG. After overnight incubation at 20 °C, cells were harvested by 5 min centrifugation at 4 °C and 6000*g* and resuspended in 50 ml of the PBS (1.8 mM KH_2_PO_4_, 140 mM NaCl, 10.1 mM NaHPO_4_, 2.7 mM KCl, pH 7.4). The bacterial suspension was sonicated and debris was removed by 1 h centrifugation at 4 °C and 50,000*g*. The supernatant was filtered through 0.22 μm polyethersulfone filter (TPP). The cell lysate was loaded onto Ni-affinity column (Ni-NTA, Qiagen), washed extensively with PBS, and eluted with a gradient of PBS with 0.5 M imidazole as a final concentration. Protein containing fractions were concentrated with Amicon Ultra 10 kDa MWCO (Merck) to a final volume of 4 ml. The protein was additionally purified with size-exclusion chromatography using Superdex 200 column (GE Healthcare) and PBS (Fig. S2*A*). Fractions of pure protein, as determined by SDS-PAGE (Fig. S2*B*), were pooled, concentrated, aliquoted and stored at −20 °C.

The genes coding mature forms of equinatoxin II (EqtII, UniProt-P61914) and fragaceatoxin C (FraC, UniProt-B9W5G6) optimized for bacterial expression were subcloned into pT7 vector, and the proteins were expressed and purified as described previously (81), with some modifications. Briefly, cells were pooled by centrifugation, resuspended in 80 ml of 20 mM phosphate buffer pH 7.2, sonicated, and centrifuged to remove debris. The filtrated cell lysate was loaded onto Sepharose Fast Flow cation exchange column (Cytiva), washed extensively with 20 mM phosphate buffer pH 7.2, and eluted in same buffer with NaCl gradient to the final 1 M NaCl concentration. Protein containing fractions were pooled and concentrated with Amicon Ultra 10 kDa MWCO (Merck) to a final volume of 4 ml. The protein was then purified with size-exclusion chromatography as described previously for bryoporin. Fractions of pure protein, as determined by SDS-PAGE (Fig. S2*B*), were pooled, concentrated, aliquoted, and stored at −20 °C.

NLP from the oomycete *Pythium aphanidermatum* (NLPPya) was prepared as described previously (68). Briefly, NLPPya complementary DNA was subcloned into the pET21c vector. Protein expression was induced with 0.5 mM IPTG for 20 h at 20 °C. NLPPya was purified by affinity chromatography (Ni–NTA agarose, Qiagen) (82), followed by gel filtration (GE Healthcare) (36).

### Protein crystallization and crystal structure determination

Bryoporin crystals were obtained by mixing 1 μl of the protein (5 mg/ml) solution with 1 μl of reservoir solution containing 2.2 M ammonium sulfate and 0.2 M NaCl using the vapor-diffusion technique in hanging drops. The drop was equilibrated at 20 °C over 0.5 ml of reservoir solution. Rod-like crystals appeared within 1 to 7 days. Crystals were frozen in liquid nitrogen, with 20% 2-methyl-2,4-pentanediol as a cryoprotectant. Diffraction data were collected at 100 K and at the wavelength of 1.0 Å at Elettra Synchrotron. The diffraction data were processed to 1.25 Å resolution with XDS (Table S1) (83). The crystal structure was solved using the symmetry of the space group P22_1_2_1_ by molecular replacement (PHASER) (84), with the crystal structure of FraC (PDB-ID 3ZWJ) without loops and α-helices as a search model. Initial bryoporin model was constructed with PHENIX Autobuild (85) and refined by iterative cycles of manual model building in Coot (86) and phenix.refine (87). Figures showing structures were prepared by PyMol (88). The crystal structure of bryoporin contains all residues except for the His_6_-tag and the first three N-terminal residues due to weak electron density in this region.

### Hemolysis assay

Bovine RBC were washed 3 to 4 times by centrifugation (800*g*) at 21 °C followed by buffer replacement. All erythrocyte buffers contained 140 mM NaCl and 20 mM buffer component, that is, Mes at pH 5.5, NaH_2_PO_4_/Na_2_HPO_4_ at pH 6.5, Tris–HCl at pH 7.4, and glycine at pH 8.5 and 9.5. After the last centrifugation, the pellet was resuspended in the corresponding buffer to reach the absorbance of ∼0.5 at 630 nm (*A*_630_), as determined with the microplate reader Synergy MX (BioTek). Hundred microliters of RBC suspension was added to each well of 96-well clear microtiter plate containing 100 μl of protein serial dilution (1:1). *A*_630_ was measured in 20 s intervals for 20 min at 25 °C. Absorbance after 20 min was normalized to the maximal value and plotted against the protein concentration. MATLAB (Mathworks) was used to fit a logistic function to the data. The midpoint value of the function was used as EC_50_.

### Lipid vesicles preparation

Lipid vesicles were produced using lipids purchased from Avanti Polar Lipids. DOPC, SM (brain, porcine), CER (brain, porcine), GlcCER (soy), CPE (brain, porcine), and sterols (cholesterol, campesterol, and ergosterol) were dissolved in chloroform at different molar ratios. Thin lipid films were generated using a rotavapor (Büchi) and left under high vacuum for 2 h. The MLVs were generated by resuspending the lipid films in appropriate buffer and thorough vortexing in the presence of 0.5 mm glass beads (Scientific Industries). MLVs suspension was then flash frozen in liquid N_2_ and subjected to at least three thaw/freeze cycles. LUVs were prepared by extrusion of MLVs with LiposoFast lipid extruder (Avestin) through polycarbonate membranes with 100 nm pores.

### SPR

SPR measurements for qualitative assessment of bryoporin interaction with membranes of different compositions were performed on Biacore X (Cytiva) at 25 °C using the L1 sensor chip (Cytiva). The running buffer was 20 mM Tris–HCl, 140 mM NaCl, 1 mM EDTA, pH 7.4. The LUV-coated chip surface was prepared as described (83, 84). After regeneration of the chip, DOPC LUVs were injected over the reference flow cell (flow cell 1), whereas LUVs of different compositions were captured on the measuring flow cell (flow cell 2) at a flow rate 2 μl/min for 10 min to 10,000 RU. This was followed by injection of 0.1 mg/ml bovine serum albumin (30 μl/min, 2 min) to prevent nonspecific binding. About 500 nM bryoporin was injected for 3 min at a flow rate of 5 μl/min and dissociation was monitored for 20 min. Data were double referenced using BIAevaluation v3.2 software (Cytiva).

SPR measurements for kinetic analysis of interactions between bryoporin or EqtII and LUVs containing either 5% SM or 5% CPE were performed using a Biacore T200 instrument (Cytiva) at 25 °C on the Series S sensor chip L1. The running buffer was PBS, pH 7.4 supplemented with 0.5% bovine serum albumin. LUVs were loaded onto the equilibrated sensor chip as described previously (69, 70). Reference flow cells (flow cells 1 and 3) contained LUVs consisting of DOPC only (as a negative control for bryoporin and EqtII binding), whereas measuring flow cells (flow cells 2 and 4) contained LUVs composed of either DOPC:CHOL:SM in a 19:19:2 molar ratio or DOPC:CHOL:CPE in a 19:19:2 M ratio. Vesicles were loaded to either ∼400 RU or ∼3500 RU. The buffer composition of the protein samples was the same as that of the running buffer. Kinetic analysis was performed with a titration of five concentrations (4-fold dilutions) of proteins injected over the LUV-coated surfaces in single-cycle mode at a flow rate of 45 μl/min with 2 min associations and dissociation was monitored for 30 or 60 min. Regeneration was performed by two consecutive 30 s injections of 40 mM octyl-β-glucoside and a mixture of isopropanol:50 mM NaOH (2:3, v:v). Blank injections were performed with the running buffer. Data were double referenced and processed using BIAevaluation v3.2.1 software (Cytiva). Sensorgrams were globally fitted to a two-state interaction kinetics model described in detail in (25, 29) and steady-state affinity analysis was used for the bryoporin DOPC:CHOL:CPE interaction. Results are presented as kinetic rates (Table S2) and *K*_*D*_s with SDs, along with a number of technical replicates.

### Calcein release assay

Calcein-loaded LUVs were prepared as described previously using 12 mM Tris–HCl, 120 mM NaCl, 50 mM calcein, and 0.5 mM EDTA (pH 7.0) buffer to dissolve thin lipid film. Excess calcein was removed from LUV suspension by gravity gel filtration on the Sephadex G-50 matrix (GE Healthcare). Concentration of DOPC and sterols was enzymatically determined with Phospholipids C kit and Free Cholesterol E kit (Wako Diagnostics), respectively. Permeabilization of 20 μM calcein-loaded LUVs by 100 nM bryoporin was measured using QuantaMaster 400 (Photon Technology International) at 485 nm and 520 nm excitation and emission wavelengths, respectively. Protein was added 200 s after the beginning of the measurement. The release of calcein was followed for 1000 s and then Triton X-100 was added to a final concentration of 2 mM to achieve 100% calcein release.

### Bryoporin binding to HeLa cells

HeLa cells were obtained from American Type Culture Collection (ATCC) and maintained in Dulbecco’s modified Eagle’s medium (DMEM) supplemented with 10% fetal calf serum, 100 units/ml penicillin, and 100 μg/ml streptomycin at 37 °C in 5% CO_2_ and 95% air incubator. Cells grown on glass coverslips were pretreated with 20 mM MβCD (Cyclolab), 1.7 unit/ml SMase from *Staphylococcus aureus* (Sigma), or DMEM/F12 medium without fetal calf serum as control at 37 °C for 30 min. The cells were then incubated with 5 μg/ml of bryoporin at 22 °C for 30 min and fixed with 4% paraformaldehyde in PBS. After blocking with 0.2% gelatine in PBS for 30 min, bryoporin was visualized with anti-His_5_ antibody (QIAGEN) and Alexa488-conjugated antimouse IgG (Thermo Fisher Scientific). The specimens were observed under an LSM510 confocal microscope (Carl Zeiss) equipped with a C-Apochromat 63XW Korr (1.2 NA) objective. The effect of MβCD and SMase was confirmed by elimination of cell staining with filipin and Eqt-II-EGFP, respectively.

### Preparation of GIPCs

GIPCs were extracted and purified from tobacco leaves (*Nicotiana tabacum*), as previously described (66, 68). Briefly, leaves were blended with cold 0.1 N aqueous acetic acid and filtered. The slurry was extracted with hot 70% ethanol/0.1 N HCl and the collected pellet was then washed with cold acetone and diethyl ether. The precipitate was dissolved in tetrahydrofuran:methanol:water (4:4:1, v:v:v) containing 0.1% formic acid, dried, and submitted to a butan-1-ol:water (1:1, v:v) phase partition. Upper butanol phase was dried and the residue was dissolved in tetrahydrofuran:methanol:water (4:4:1, v:v:v) containing 0.1% formic acid. GIPCs were characterized by MALDI-MS (66) and their mass was estimated from dry weight.

### Lipid sedimentation assay

Lipid sedimentation assay was performed as previously described (68). Briefly, proteins (0.0625 mg/ml) were incubated with MLVs (5 mM) in PBS for 30 min at 600 rpm and room temperature. The control experiments contained PBS instead of MLVs. The mixture was centrifuged (30 min at 16,100*g*), and the supernatant was removed and stored for later analysis with SDS-PAGE. The pellet was washed twice with PBS and collected with centrifugation (20 min at 16,100*g*). Pellet and supernatant were subjected to SDS-PAGE, followed by Coomassie Brilliant Blue staining. The ratio between the bands of pellet and supernatant was quantified with GelQuant.NET software provided by biochemlabsolutions.com. Ratios of bound protein of at least four experiments were averaged and plotted.

### Leaf infiltration assay

The experiments were performed as described in (68). Briefly, adult tobacco (*N. tabacum*) leaves were infiltrated abaxially with 1 or 10 μM solutions of bryoporin, EqtII, FraC, and NLP_Pya_ or corresponding buffers (*i.e.*, PBS for bryoporin, EqtII, and FraC and 20 mM Mes, 50 mM NaCl, pH 5.8 for NLP_Pya_) using a needleless syringe. Necrotic symptoms occurred within 1 to 2 h, and images were taken after 48 h to enhance the visibility of the phenotype. Experiments were performed on three different leaves from three different plants.

### Planar lipid membranes experiments

For electrical measurements in planar lipid bilayers an integrated chip-based recording setup Orbit mini and EDR3 software (Nanion Technologies) were used. Recordings were obtained in parallel with multielectrode cavity array chips (Meca 4150 μm, Ionera Technologies). Lipid bilayers were formed by painting 10 mg/ml solution of DOPC and SM (molar ratio 1:1) dissolved in octane over the microcavity. The electrolyte solution was 0.5 M NaCl, 20 mM Mes, pH 6. Bryoporin was added to the open cavity of the chip (the *cis* side of the bilayer). Alternating voltages of −50 mV and 50 mV were applied. Sampling rate was 20 kHz. Current traces were analyzed using Clampfit 10.6 (Molecular Devices) and MATLAB r2016b (The MathWorks).

### Negative staining TEM

MLVs were prepared in PBS, as described previously. Bryoporin was added to MLVs and the mixture was incubated for 30 min at 37 °C. The final concentration of bryoporin and MLVs was 500 nM and 10 mM, respectively. Copper mesh grids (SPI Supplies) were Formvar coated, stabilized with carbon, and glow discharged (EM ACE200, Leica Microsystems). The MLVs protein suspension (2 μl) was applied to a grid and contrasted with 1% uranyl acetate (aqueous solution). Samples were imaged at 80 kV by CM 100 transmission electron microscope (Philips), equipped with Orius SC 200 camera (Gatan), and Digital Micrograph software.

### TEM at cryogenic conditions (cryo-EM)

Sample of bryoporin with MLVs was prepared as described previously for negative staining TEM. Three microliters of suspension was applied to a glow discharged (GloQube Plus, Quorum) Quantifoil R1.2/1.3300-mesh copper holey carbon grid (Quantifoil), blotted under 100% humidity at 4 °C for 6 to 7 s, and plunged into liquid ethane using a Mark IV Vitrobot (Thermo Fisher Scientific). Micrographs were collected on cryo-transmission electron microscope (Glacios, Thermo Fisher Scientific) with a Falcon 3EC direct electron detector (Thermo Fisher Scientific) and operated at 200 kV using the EPU software (Thermo Fisher Scientific). Images were recorded in counting mode with the pixel size of 1.6 Å. Micrographs were dose fractioned into 38 frames with total dose of 30 e−/Å.

### Cryo-EM image processing

All steps of data processing were performed in cryoSPARC 2.4 (89) with built-in algorithms. Cryo-EM data were analyzed following the steps of a typical single particle analysis protocol stopping at 2D classification. Micrographs were dose weighted and motion corrected. After contrast transfer function estimation, 1000 particles were handpicked and underwent 2D classifications to create 2D class averages.

### Bryoporin pore modeling

The structure of the monomeric bryoporin in combination with stoichiometry information obtained by cryo-EM was used to model the bryoporin pore. The shape of the bryoporin pore could not be reconstructed only by including copies of the monomeric bryoporin. Since the shape of the bryoporin pore is highly similar to that of FraC pore (PDB-ID 4TSY) (26), we used the FraC pore structure as a template. The elongated membrane spanning N-terminal α-helix of each protomer was modeled using SWISS-MODEL (90, 91, 92, 93, 94), employing the amino acid sequence of bryoporin from Met1 to the Gly25 (Fig. 1*A*) and the corresponding region of the FraC protomer (PDB-ID 4TSY) as a structural template. The rest of the protomer structure remained unchanged in comparison to the monomeric bryoporin (Fig. 6*D*). Finally, eight bryoporin protomers were aligned to FraC pore structure.

## Data availability

The data that support the findings of this study are available from the corresponding author upon request. The crystal structure of bryoporin was deposited at the Protein Data Bank (https://www.rcsb.org/) under PDB-ID code 7PUD.

## Supporting information

This article contains supporting information (26, 95, 96, 97, 98).

## Conflict of interest

The authors declare that they have no conflicts of interest with the contents of this article.
