# Supplementary material for: Pore-forming moss protein bryoporin is structurally and mechanistically related to actinoporins from evolutionarily distant cnidarians
Source: J Biol Chem. 2022 Sep 3;298(10):102455. doi: 10.1016/j.jbc.2022.102455 (PMC9526159; doi:10.1016/j.jbc.2022.102455)
Supplement: Supplemental Figures S1–S9 and Tables S1, S2 [file mmc1.docx]

**Pore-forming moss protein bryoporin is structurally and mechanistically related to actinoporins from evolutionarily distant cnidarians**

Gašper Šolinc, Tomaž Švigelj, Neža Omersa, Tina Snoj, Katja Pirc, Nada Žnidaršič, Akiko Yamaji-Hasegawa, Toshihide Kobayashi, Gregor Anderluh and Marjetka Podobnik

**Supporting Information**

[Figure S1 **Phylogenetic relationships of actinoporins** S-2](#_Toc104970811)

[Figure S2 **Purity of proteins used in this study** S-3](#_Toc104970812)

[Figure S3 **Electron density map of a segment in the crystal structure of bryoporin** S-4](#_Toc104970813)

[Figure S4 **Structural comparison of bryoporin and HALT1** S-5](#_Toc104970814)

[Figure S5 **FraC monomer with two lipid molecules bound to it, superimposed with bryoporin** S-6](#_Toc104970815)

[Figure S6 **Comparison of electrostatic surface potentials of monomeric bryoporin and monomeric FraC** S-7](#_Toc104970816)

[Figure S7 **Selected examples of SDS-PAGE gels used in analysis of bryoporin binding to multilamellar vesicles by the lipid sedimentation assay** S-8](#_Toc104970817)

[Figure S8 **The effect of proteins on formation of necrotic lesions in tobacco leaves** S-9](#_Toc104970818)

[Figure S9 **Structures of sphingolipids** S-10](#_Toc104970819)

[Table S1 **X-ray diffraction data collection and crystallographic refinement statistics** S-11](#_Toc101270817)

Table S2 **Rate and affinity constants for the binding of bryoporin and EqtII to large unilamellar vesicles**.……………………………………………………………S-12


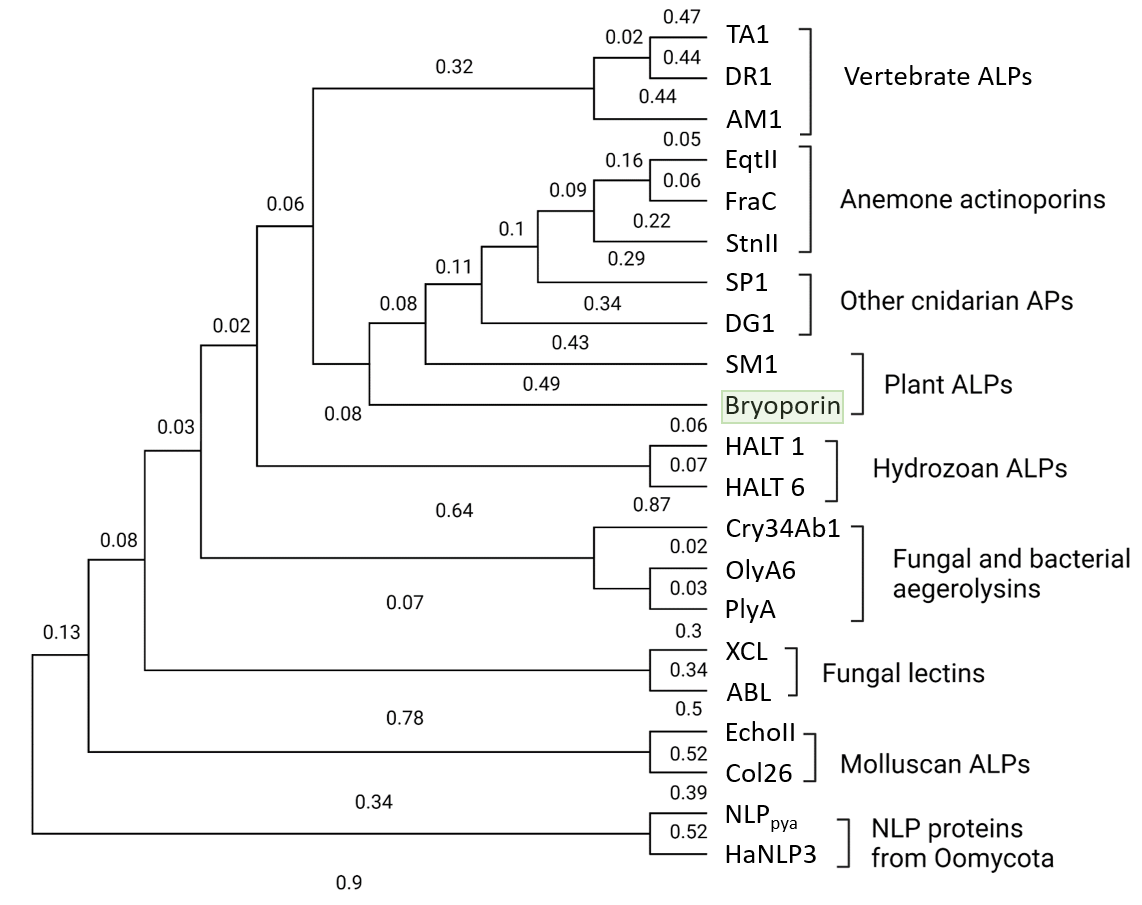


Figure S1 **Phylogenetic relationships of actinoporins.** The evolutionary history was inferred using the Neighbor-Joining method (1). The optimal tree is shown. The evolutionary distances were computed using the Poisson correction method (2) and are in the units of the number of amino acid substitutions per site. This analysis involved 21 amino acid sequences. Evolutionary analyses were conducted in MEGA11 (3). Abbreviations of the protein, origin species names with database and appropriate identification numbers in brackets are as follows: TA1, *Thunnus albacares* (NCBI: XP_044187674.1); DR1, *Danio rerio* (NCBI: NP_001280600.1); AM1, *Astyanax mexicanus* (GenBank: KAG9268248.1); EqtII, equinatoxin II from *Actinia equine* (UniProt: P61914); FraC, fragaceatoxin C from *Actinia fragacea* (UniProt: B9W5G6); StnII, sticholysin II form *Stichodactyla helianthusm* (UniProt: P07845); SP1, *Stylophora pistillata* (NCBI: XP_022777760.1); DG1, *Dendronephthya gigantean* (NCBI: XP_028397903.1); SM1, *Selaginella moellendorffii* (GenBank: EFJ24276.1); Bryoporin form *Physcomitrella patens* (UniProt: Q5UCA8); HALT (Hydra actinoporin like toxin) 1 (NCBI: XP_012562760.2) and 6 (NCBI: XP_012562488.1) from *Hydra vulgaris*; Cry34Ab1 from *Bacillus thuringiensis* (UniProt: Q939T0); OlyA6, Ostreolysin A from *Pleurotus ostreatus* (UniProt: P83467); PlyA , pleurotolysin A from *Pleurotus eryngii* (UniProt: Q8X1M9); XCL, lectin from *Xerocomus chrysenteron* (GenBank: AAL73235.1); ABL, lectin from *Agaricus bisporus* (UniProt: Q00022.3); Col26, coluporin-26 from *Colubraria reticulata* (GenGank: AXS67898.1); EchoII, Echotoxin-2 from *Monoplex parthenopeus* (*Monoplex echo*) (UniProt: Q76CA2 ) NLPpya, NEP-like protein from *Pythium aphanidermatum* (GenBank: AAD53944.1); HaNLP3, NEP-like protein from *Hyaloperonospora arabidopsidis* (GenBank: AEZ06577.1).


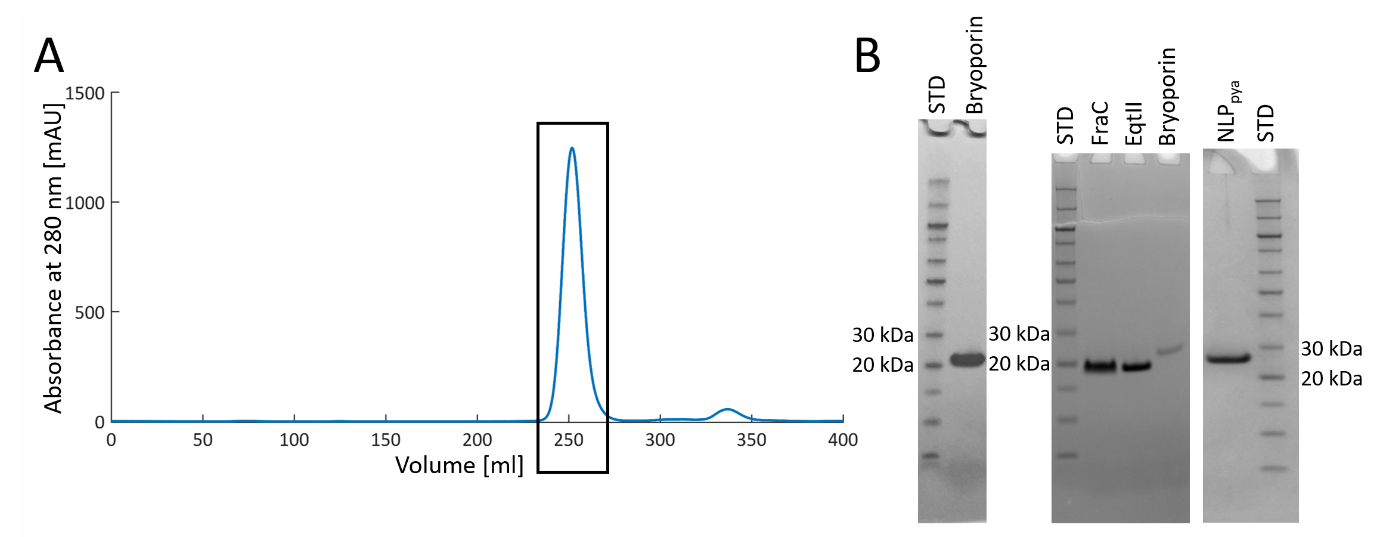


Figure S2 **Purity of proteins used in this study.** A, Chromatogram of bryoporin injected to Superdex 200 26/60 column. The highlighted peak (black rectangle) was concentrated and applied to SDS-PAGE shown in B. Elution volume of 250ml of the peak corresponds to the size of monomeric bryoporin according to Gel Filtration Calibration Kit (GE Healthcare, USA). B, SDS-PAGE gels of actinoporins. STD, molecular weight standard (Novex™ Sharp Unstained, Thermo Fisher Scientific, USA). FraC, fragaceatoxin C; EqtII, equinatoxin II; NLP_pya_ Necrosis and ethylene inducing (Nep1)-like protein from *Pythium aphanidermatum.*


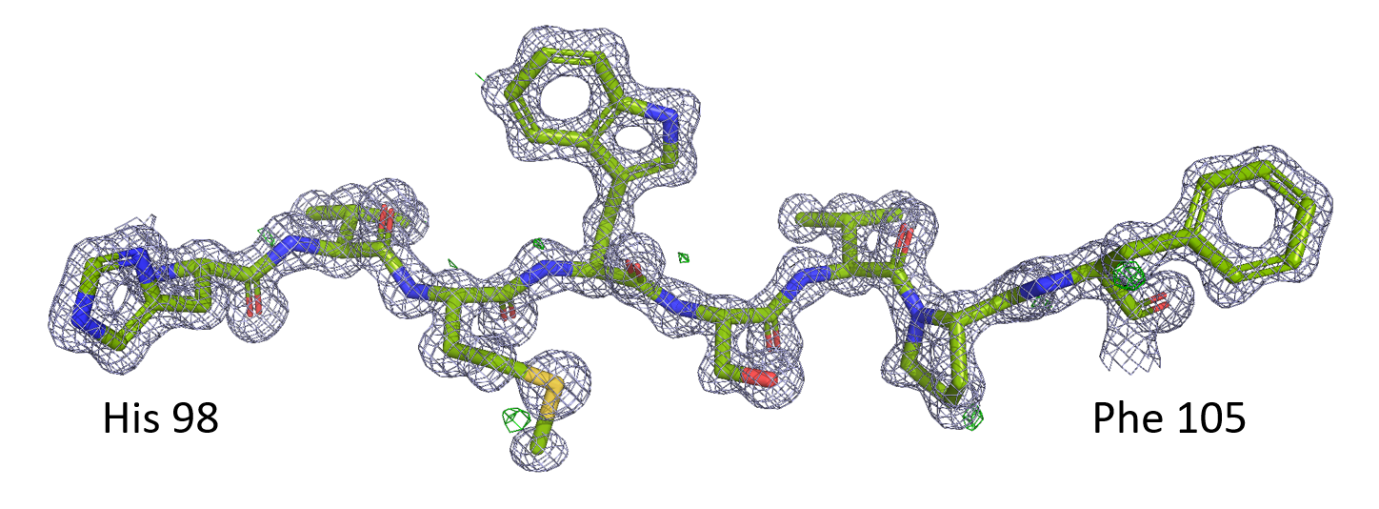


Figure S3 **Electron density map of a segment in the crystal structure of bryoporin.** Bryoporin region His98 - Phe105 and corresponding electron density maps are displayed. 2mFo-DFc electron density is contoured at 3 σ (gray) and mFo-DFc electron density at +3.0 σ (green) and at -3.0 σ (red).


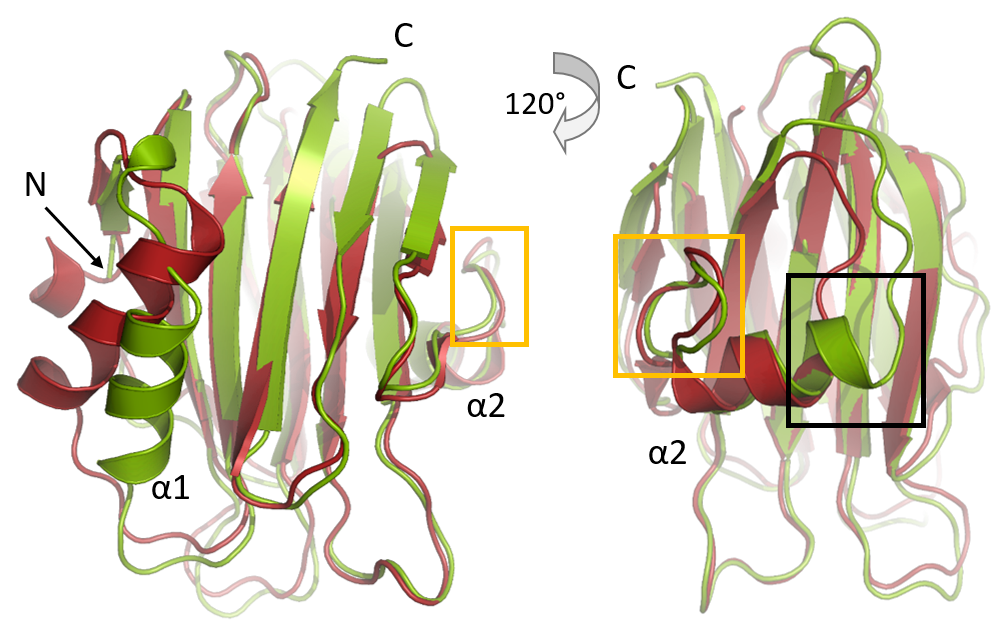


Figure S4 **Structural comparison of bryoporin (green, PDB-ID 7PUD) and HALT1 (red, PDB-ID 7EKZ).** N- and C-termini and α-helices are labelled. The orange rectangle marks the position of the extra loop in bryoporin and HALT-1, which is not present in cnidarian actinoporins. The differences in the length of α2-helix between the two proteins is marked with a black rectangle.


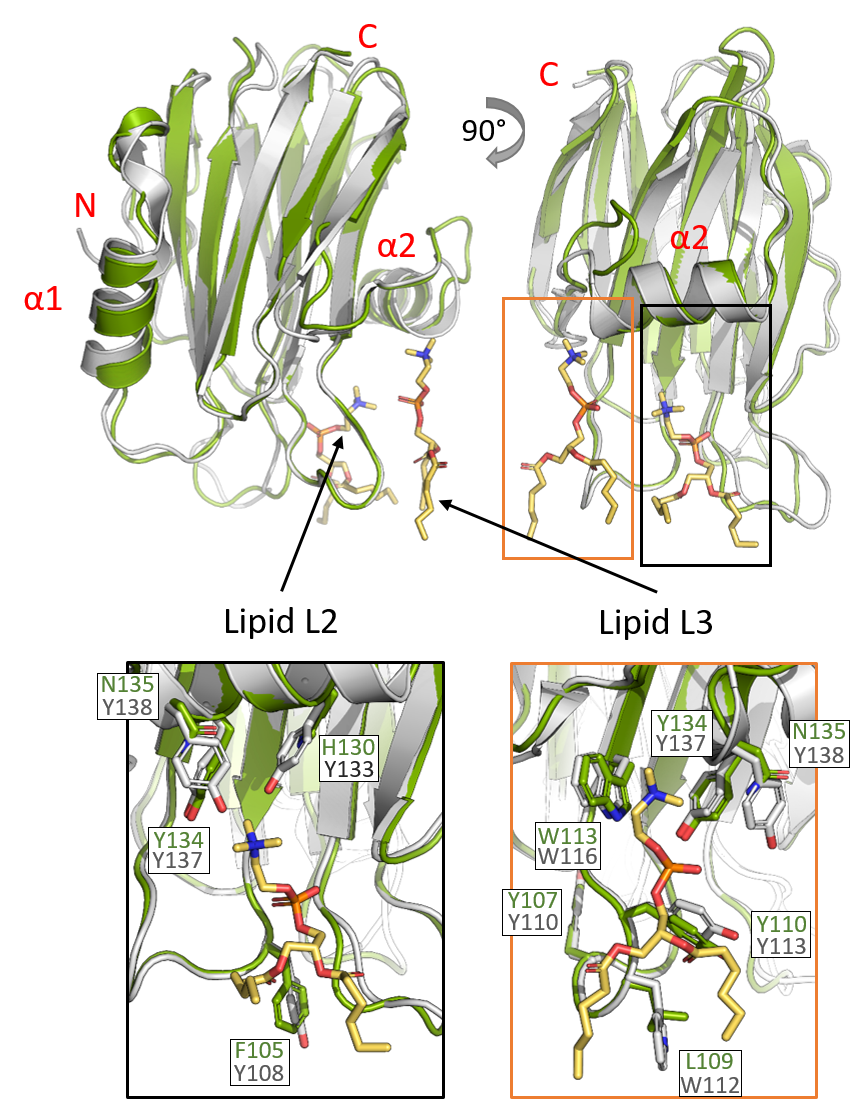


Figure S5 **FraC monomer with two lipid molecules bound to it (gray, PDB-ID 4TSO), superimposed with bryoporin (green, this study, PDB-ID 7PUD).** Two lipid molecules (1,2-dihexanoyl-*sn*-glycero-3-phosphocholine (DHPC)) from FraC structure are shown as yellow sticks and labeled as Lipid L2 (black rectangle) and Lipid L3 (orange rectangle), as per the original paper (4). The residues involved in lipid-binding are highlighted for each lipid. N- and C-termini and α-helices are labelled.


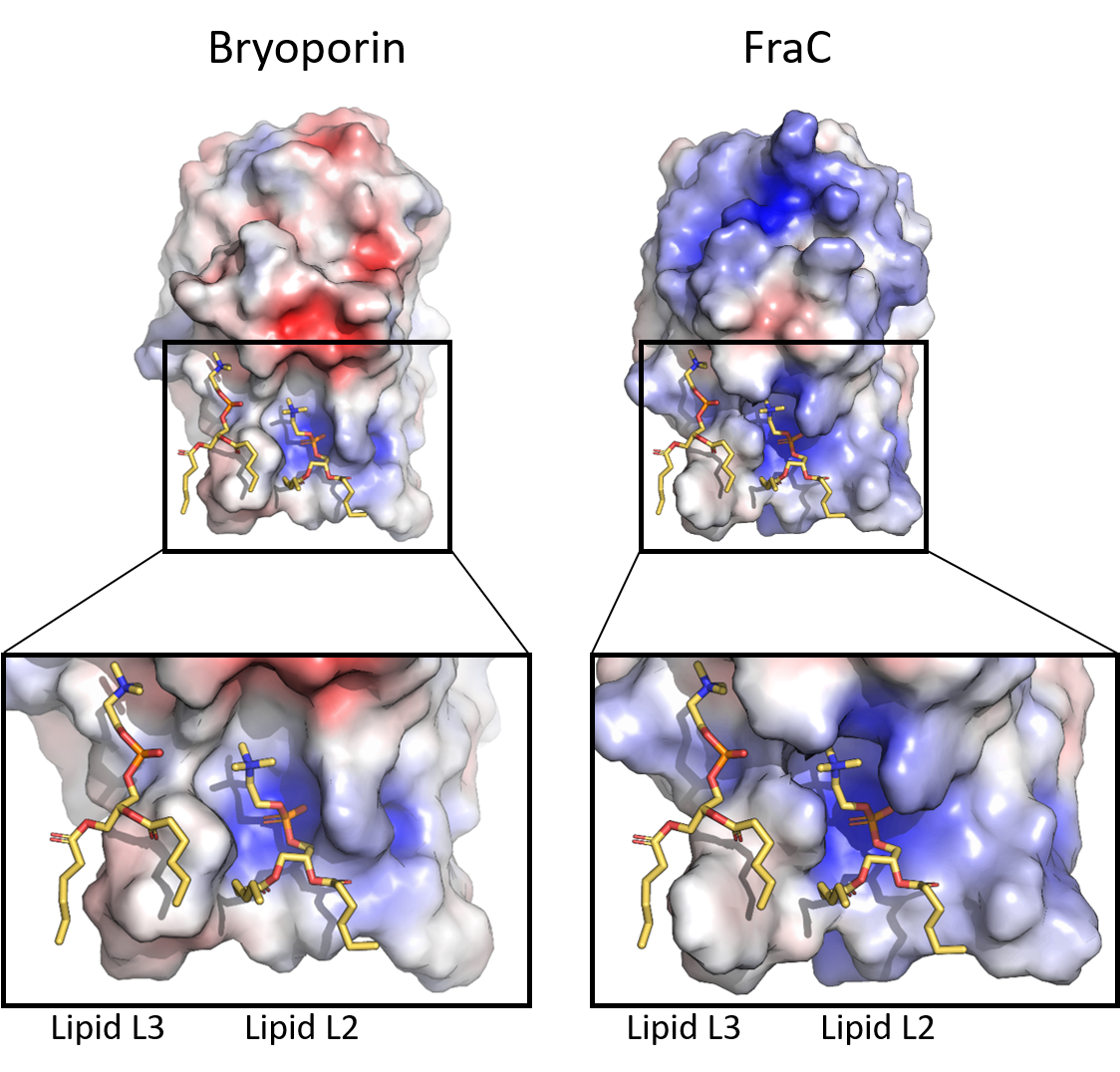


Figure S6 **Comparison of electrostatic surface potentials of the monomeric bryoporin (left, PDB-ID 7PUD) and monomeric FraC (right, PDB-ID 4TSO).** Two molecules of 1,2-dihexanoyl-*sn*-glycero-3-phosphocholine (DHPC) lipid bound to FraC are shown in yellow sticks and labeled Lipid L2 and Lipid L3, as per the original paper (4). The electrostatic surface potential was calculated with APBS plugin in PyMol (5) (red −5 kbT/ec, blue +5 kbT/ec). The area where DHPC molecules are bound in FraC monomer structure (PDB-ID 4TSO) and the equivalent position in bryoporin is zoomed in.


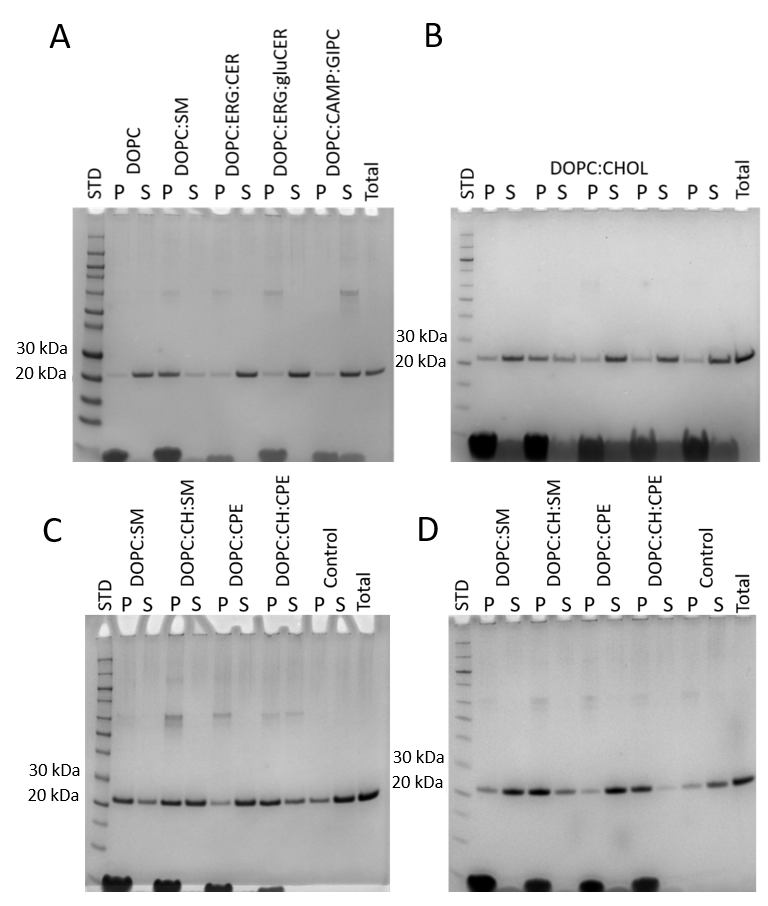


Figure S7 **Selected examples of SDS-PAGE gels used in analysis of bryoporin binding to multilamellar vesicles by the lipid sedimentation assay.** A, Molar ratios of lipids are 2:1 and 1:1:1 for DOPC:SM and three-component lipid vesicles, respectively. B, DOPC:CHOL lipids in vesicles are in 2:1 molar ratio. C, Molar ratios of lipids are 2:1 for two-component lipid vesicles, and 1:1:1 for three-component lipid vesicles. D, Molar ratios of lipids are 19:1 for two-component systems, and 19:19:2 for three-component vesicles (i.e. 5% of SM or CPE). STD, molecular weight standard (Novex™ Sharp Unstained, Thermo Fisher Scientific, USA); P, pellet; S, supernatant; Total, total amount of protein used in experiment (i.e. 1 µg); Control, protein without lipid vesicles. Experiments were repeated four or six times. The % of protein binding is shown on Fig. 3*B*.


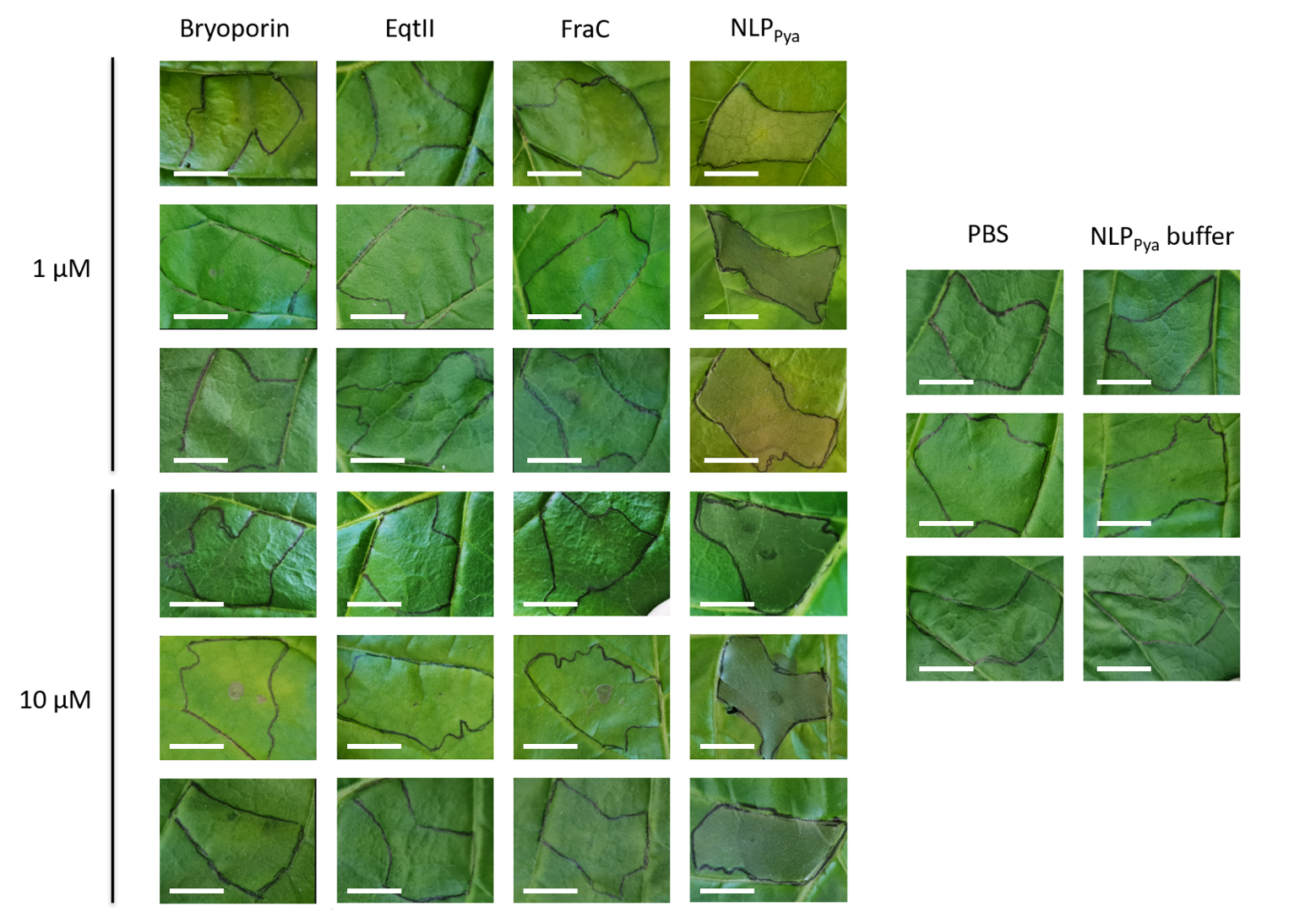


Figure S8 **The effect of proteins on formation of necrotic lesions in tobacco leaves.** Tobacco leaves were infiltrated with 1 or 10 µM solutions of bryoporin, EqtII, FraC in PBS and NLP_Pya_ in MES buffer. Each experiment was repeated three times on a different leaf from a different plant. The infiltrated areas are marked. The upper surface of the leaf was photographed after 48 hours. Scale bar is 1 cm. Representative pictures are shown in Fig. 3*C*.


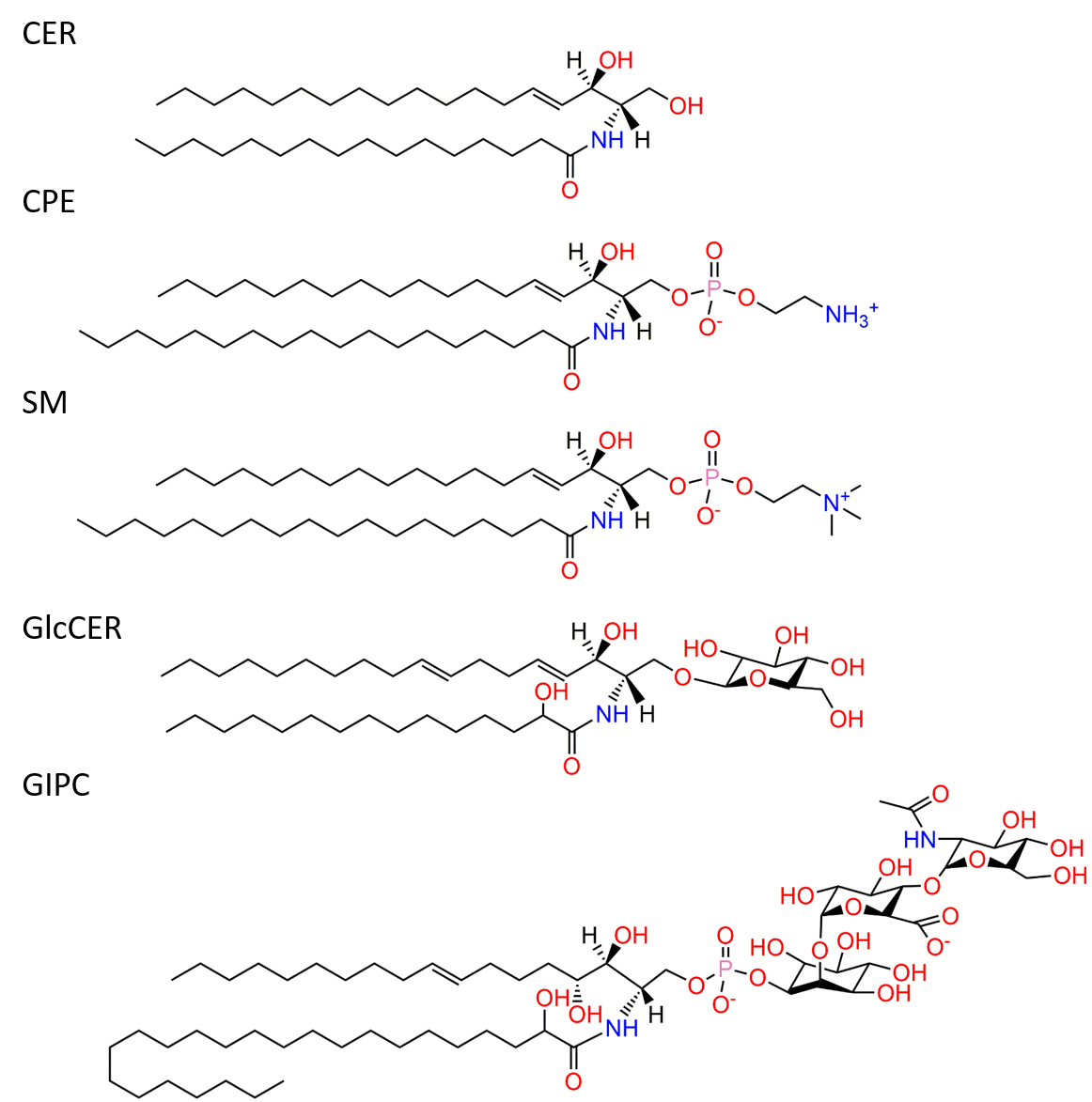


Figure S9 **Structures of sphingolipids**. CER, ceramide; CPE, ceramide phosphoethanolamine; SM, sphingomyelin; GlcCER, glucosylceramide; GIPC, glycosylinositol phosphorylceramide.

Table S1 **X-ray diffraction data collection and crystallographic refinement statistics.**

| **Data collection** | | **Bryoporin** |
| --- | --- | --- |
| Resolution range (Å) | | 32.75 - 1.25 |
| Space group | | P 2 2_1_ 2_1_ |
| Unit cell dimensions | |  |
| a, b, c (Å) | | 37.92, 54.45, 81.97 |
| α, β, γ (°) | | 90, 90, 90 |
| Total reflections | | 279525 (20335) |
| Unique reflections | | 46462 (3873) |
| Multiplicity | | 6.0 (4.9) |
| Completeness (%) | | 93.50 (83.35) |
| Mean I/sigma(I) | | 21.02 (3.62) |
| R_merge_ | | 0.04733 (0.4168) |
| R_meas_ | | 0.05227 (0.465) |
| R_pim_ | | 0.02163 (0.2023) |
| CC_1/2_ | | 0.999 (0.933) |
| **Refinement** | |  |
| Reflections used in refinement | | 44669 (3874) |
| Reflections used for R-free | | 1972 (172) |
| R-work (%) | | 21.06 (33.92) |
| R-free (%) | | 23.36 (36.51) |
| Number of non-hydrogen atoms | | 1692 |
| Protein | | 1437 |
| Ligands | | 56 |
| MPD | 16 |  |
| SO_4_^2-^ | 40 |  |
| Water | | 182 |
| Protein residues | | 175 |
| RMS deviations - bonds(Å) | | 0.006 |
| RMS deviations - angles (°) | | 0.85 |
| Ramachandran plot | |  |
| Favored (%) | | 97.69 |
| Allowed (%) | | 2.31 |
| Outliers (%) | | 0.00 |
| Rotamer outliers (%) | | 0.62 |
| Clash score | | 2.69 |
| Average B-factor (Å^2^) | | 14.67 |
| Protein | | 12.71 |
| Ligands | |  |
| MPD | | 26.16 |
| SO_4_^2-^ | | 21.20 |
| H_2_O | | 25.79 |

One crystal was used to collect the data. There was one molecule of bryoporin in the asymmetric unit. Values in parentheses are for the highest-resolution shell.

Table S2: **Rate and affinity constants for the binding of bryoporin and EqtII to large unilamellar vesicles (LUVs)**. Rate constants were determined from sensorgrams recorded for protein binding to LUVs composed of DOPC:CHOL:SM or DOPC:CHOL:CPE in 19:19:2 molar ratio. A two-state kinetic model was used to fit the experimental data, except in the case of bryoporin binding to DOPC:CHOL:CPE. Here, because of very fast association and dissociation phases, a steady state affinity model was used instead (Fig. 4*B*). Bryoporin concentrations did not reach the calculated K_D_ value which is therefore not accurate and reported in the table as not available (na). Average values ± standard deviation are shown. N is the number of technical replicates.

| **Protein** | **Lipids**  (19:19:2 mol. ratio) | ***k*_a1_**  × 10^5^ [M^-1^s^-1^] | ***k*_d1_**  × 10^-3^ [s^-1^] | ***k*_a2_**  × 10^-3^ [s^-1^] | ***k*_d2_**  × 10^-3^ [s^-1^] | ***K_D_***  × 10^-9^ [M] | **N** |
| --- | --- | --- | --- | --- | --- | --- | --- |
| Bryoporin | DOPC:CHOL:SM | 7.02±1.48 | 34.04±2.72 | 0.51±0.12 | 0.52±0.11 | 24.85±3.33 | 6 |
|  | DOPC:CHOL:CPE | na | na | na | na | na | 5 |
| EqtII | DOPC:CHOL:SM | 16.83±6.97 | 7.07±5.87 | 6.48±6.40 | 1.40± 2.18 | 0.09±0.09 | 6 |
|  | DOPC:CHOL:CPE | 7.24±3.69 | 115.68±43.69 | 0.64±0.38 | 0.67±0.10 | 90.99±26.91 | 8 |

**References**

1. Saitou, N., and Nei, M. (1987) The neighbor-joining method: a new method for reconstructing phylogenetic trees. *Mol. Biol. Evol.* **4**, 406–425

2. Zuckerkandl, E., and Pauling, L. (1965) Evolutionary divergence and convergence in proteins. *Evol. Genes Proteins*. 10.1016/B978-1-4832-2734-4.50017-6

3. Tamura, K., Stecher, G., and Kumar, S. (2021) MEGA11: molecular evolutionary genetics analysis version 11. *Mol. Biol. Evol.* **38**, 3022–3027

4. Tanaka, K., Caaveiro, J. M. M., Morante, K., González-Manãs, J. M., and Tsumoto, K. (2015) Structural basis for self-assembly of a cytolytic pore lined by protein and lipid. Nat. Commun. **10**.1038/ncomms7337

5. Jurrus, E., Engel, D., Star, K., Monson, K., Brandi, J., Felberg, L. E., Brookes, D. H., Wilson, L., Chen, J., Liles, K., Chun, M., Li, P., Gohara, D. W., Dolinsky, T., Konecny, R., Koes, D. R., Nielsen, J. E., Head-Gordon, T., Geng, W., Krasny, R., Wei, G. W., Holst, M. J., McCammon, J. A., and Baker, N. A. (2018) Improvements to the APBS biomolecular solvation software suite. Protein Sci. **27**, 112–128
